# Structural dynamics of receptor recognition and pH-induced dissociation of full-length *Clostridioides difficile* Toxin B

**DOI:** 10.1371/journal.pbio.3001589

**Published:** 2022-03-24

**Authors:** Mengqiu Jiang, Joonyoung Shin, Rudo Simeon, Jeng-Yih Chang, Ran Meng, Yuhang Wang, Omkar Shinde, Pingwei Li, Zhilei Chen, Junjie Zhang

**Affiliations:** 1 Department of Biochemistry and Biophysics, Texas A&M University, College Station, Texas, United States of America; 2 Department of Microbial Pathogenesis and Immunology, Texas A&M University Health Science Center, College Station, Texas, United States of America; Georgia Institute of Technology, UNITED STATES

## Abstract

*Clostridioides difficile* secretes Toxin B (TcdB) as one of its major virulence factors, which binds to intestinal epithelial and subepithelial receptors, including frizzled proteins and chondroitin sulfate proteoglycan 4 (CSPG4). Here, we present cryo-EM structures of full-length TcdB in complex with the CSPG4 domain 1 fragment (D1_401-560_) at cytosolic pH and the cysteine-rich domain of frizzled-2 (CRD2) at both cytosolic and acidic pHs. CSPG4 specifically binds to the autoprocessing and delivery domains of TcdB via networks of salt bridges, hydrophobic and aromatic/proline interactions, which are disrupted upon acidification eventually leading to CSPG4 drastically dissociating from TcdB. In contrast, FZD2 moderately dissociates from TcdB under acidic pH, most likely due to its partial unfolding. These results reveal structural dynamics of TcdB during its preentry step upon endosomal acidification, which provide a basis for developing therapeutics against *C*. *difficile* infections.

## Abstract 2

Clostridioides difficile secretes Toxin B (TcdB) as one of its major virulence factors, which binds to intestinal receptors. This structural study of TcdB in complex with frizzled-2 and chondroitin sulfate proteoglycan 4 reveals how TcdB binds to human receptors and primes itself for host entry.

## Introduction

**Fig 1:**
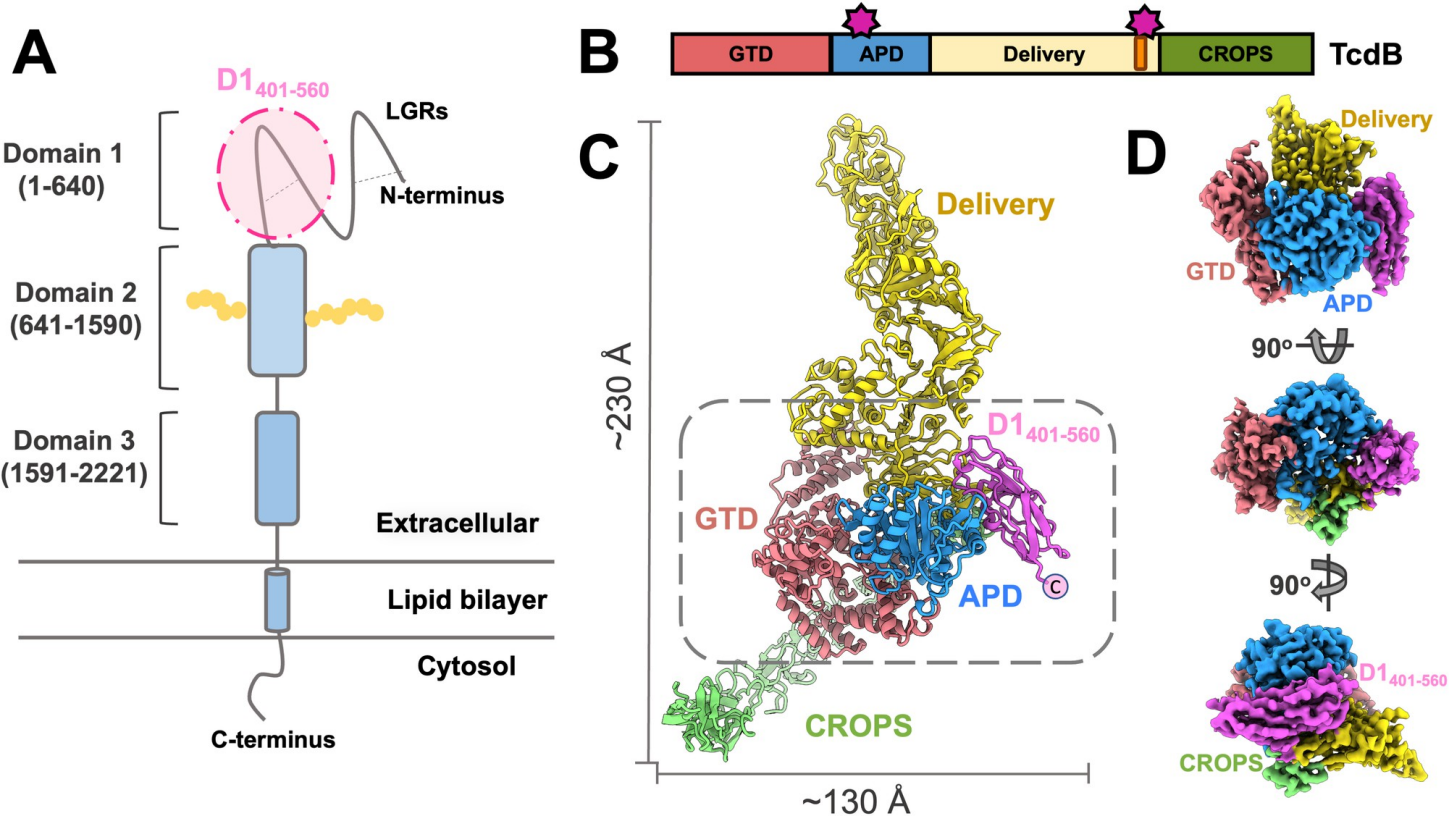
The overall structure of the complex between TcdB and D1_401-560_. (A) The scheme for the domain organization of CSPG4. The 3 domains of CSPG4 are shown in a cartoon style, with their corresponding residues labeled. Yellow connected dots represent glycosylation on Domain 2. D1_401-560_ is highlighted by a pink oval. (B) The color scheme of TcdB domains. Regions, which bind D1_401-560_, are labeled by magenta stars. The D97 region is labeled orange. (C) The model of the full-length TcdB-D1_401-560_ complex, with domains labeled and colored based on the scheme in Panel B. The dashed gray box indicates the central region for the focused cryo-EM map refinement. The dimensions of the complex are labeled. (D) The density map of the central region, viewed from different angles, with each domain color coded. APD, autoprocessing domain; CROPS, combined repetitive oligopeptides; CSPG4, chondroitin sulfate proteoglycan 4; GTD, glucosyltransferase domain; LGR, laminin G-type region; TcdB, Toxin B.

*Clostridioides difficile* is an anaerobic, spore-forming bacterium, responsible for most nosocomial infections worldwide. In the United States, *C*. *difficile* infections (CDIs) cost the healthcare system $4.8 billion annually [1]. Toxin B (TcdB) is one of the major virulence factors of *C*. *difficile*, which causes inflammation leading to symptoms ranging from diarrhea to life-threatening colitis [2]. TcdB binds to the receptors on the surface of the cells and enters the cell through endocytosis [3]. It consists of 4 domains (**Fig 1B**): a glucosyltransferase domain (GTD, residues 1 to 543), an autoprocessing domain (APD, residues 544 to 841), a delivery domain (residues 842 to 1,834), and a combined repetitive oligopeptides (CROPS) domain (residues 1,835 to 2,366). The delivery domain (residues 842 to 1,834) makes up the skeleton of TcdB and contains a pore-forming region, which undergoes a conformational change upon acidification in late endosomes [4,5]. This conformational change delivers both GTD and APD across the endosomal membrane into the cytosol by allowing cytosolic inositol hexakisphosphate (IP_6_) to activate the APD, which cleaves and releases the GTD [6,7]. The GTD then glucosylates small GTPases in host cells, irreversibly altering cellular activities [8]. The CROPS domain was proposed to be involved in receptor binding as well as regulation of the APD activity [9,10]. Recently, it has been shown that the delivery domain participated in binding host receptors, such as frizzled proteins and the chondroitin sulfate proteoglycan 4 (CSPG4) [11,12]. Frizzled proteins are a family of receptors involved in signaling pathways, such as the Wnt signaling [13]. A recent crystal structure of a TcdB fragment in complex with the cysteine-rich domain (CRD) of human frizzled-2 (FZD2) protein revealed the structural basis for the recognition of frizzled proteins by TcdB [14]. The study proposed that an endogenous FZD-bound fatty acid acts as a coreceptor for TcdB binding.

CSPG4 is a highly glycosylated membrane protein and is implicated in cellular signaling pathways, such as the mitogen-activated protein kinase pathway and the focal adhesion kinase (FAK) pathway [15]. In many cancer cell lines, the expression level of CSPG4 is significantly higher, suggesting a potential new target for the antibody-based immunotherapy of cancers [16,17]. As CSPG4 is expressed in the subepithelial layer of the intestine, it facilitates deeper penetration of TcdB into intestinal tissues [12]. Cells that express both CSPG4 and frizzled proteins are more susceptible to TcdB toxicity than the cells that only express frizzled proteins [18], favoring a “dual-receptor” model, in which the binding of CSPG4 or frizzled proteins enhances the uptake efficiency of TcdB.

The large extracellular region (approximately 220 kD, residues 1 to 2,221) of CSPG4 can be divided into 3 domains [19] (**Fig 1A**): Domain 1 (D1, residues 1 to 640) has the first 400 amino acids folded into 2 laminin G-type regions (LGRs), followed by a C-terminal region (residues 401 to 560, D1_401-560_). D1 is involved in ligand binding, integrin interactions, and cell–cell connections [20]. Disulfide bonds within D1 stabilize the tertiary structures. Domain 2 (D2, residues 641 to 1,590) consists of 15 repetitive “CSPG” motifs and has been hypothesized to interact with growth factors. Domain 3 (D3, residues 1,591 to 2,221) is a globular domain that is proximal to the cell membrane and binds lectins [21].

D1 was proposed to interact with TcdB with high affinity, and the binding site was located in residues 401 to 560 (D1_401-560_) [12]. In addition, truncations of TcdB were used to locate the CSPG4-binding site to be within residues 1,500 to 1,851 of TcdB. Recently, there is a published cryo-EM structure of the complex between a TcdB fragment and CSPG4 [22]. However, the structural mechanism of how the full-length TcdB binds CSPG4 is unclear. During endocytosis, TcdB is proposed to undergo a conformational change upon endosomal acidification in the late stage of endocytosis [23]. Impact of acidification on receptor binding to TcdB remains unknown.

Here, we present high-resolution cryo-EM structures of the full-length TcdB with the TcdB-binding domains of CSPG4 and FZD2, respectively. Our results reveal that the C-terminal fragment of CSPG4 D1 (D1_401-560_) adopts a barrel shape and forms tight interactions with TcdB via electrostatic, hydrophobic and aromatic/proline interactions. These interactions are confirmed by site-directed mutagenesis. Furthermore, we see that while most D1_401-560_ of CSPG4 dissociate from TcdB upon acidification, a significant fraction of the CRD of FZD2 still binds to TcdB, albeit in a loosely bound fashion. Such a difference of CSPG4 and FZD2 in binding TcdB upon acidification is consistent with the different sizes of the extracellular domains for each receptor and their different binding sites on TcdB. This may allow the repositioning of TcdB to bring its pore-forming helices proximal to the endosomal membrane, facilitating the translocation of effector domains from inside the endosomal lumen to the cytosol.

## Results

### The overall structure of the full-length TcdB in complex with D1_401-560_ at cytosolic pH

We conduct a single-particle cryo-EM analysis on the protein complex between TcdB and D1_401-560_. The complete cryo-EM density for the complex between the full-length TcdB and D1_401-560_ is resolved to 4.8 Å resolution. The entire complex measures approximately 230 Å in length and approximately 130 Å in width, with TcdB presented in an extended conformation, in which the delivery and CROPS domains protrude toward opposite directions. The overall resolution of the complete cryo-EM density map is limited by the flexibility in the delivery and CROPS domains (**S1 Fig**). Therefore, to further reveal the molecular details at the interface between TcdB and D1_401-560_, we focused our image processing on the central region of the complex, including D1_401-560_, GTD, APD, and part of the delivery and CROPS domains. A 3.4-Å resolution density map of this local region was reconstructed with clear densities for polypeptide backbone and bulky side chains (**S1 Fig**), enabling us to build an atomic model of the complex between TcdB and D1_401-560_. The residues from 401 to 409 were not visible in the density map, possibly because of the flexibility at the N-terminus of this CSPG4 fragment. D1_401-560_ adopts a β-barrel shape and is clamped in the middle of TcdB at the opposite side of GTD by the N-terminal region of APD and the C-terminal region of the delivery domain (**Fig 1B–1E**). Notably, the “D97” region (97 residues from Glu1756 to Leu1852 in the delivery domain, labeled gold in **Fig 1B**), contains one of the binding sites and was previously proposed to be functionally important to the toxicity of TcdB [24]. While one end of the β-barrel, which includes the C-terminus (labeled in **Fig 1C**), is surface exposed, the other end of the β-barrel is engaged in the interactions with TcdB.

The β-barrel of D1_401-560_ is formed by 6 β-strands (β1–6) connected by loops and 5 short α-helices (α1–5) (**S2 Fig**). Such a β-barrel structure of D1_401-560_ provides a stable structural scaffold, from which the connecting loops and α-helices present residues to interact with TcdB. D1_401-560_ measures 50 Å × 30 Å × 20 Å in dimensions (**S2 Fig**) and binds to TcdB with the longest axis parallel to the cleft formed by APD, the C-terminal region of the delivery domain, and the N-terminus of the CROPS (**Fig 1D**). More specifically, APD of TcdB interacts with α2 and the loop connecting α2–3 in D1_401-560_ (labeled by blue stars in **S2 Fig**); and the delivery domain of TcdB interacts with the loop connecting β-strands 3–4 (labeled by a yellow star in **S2 Fig**). The N- and C-terminus of D1_401-560_ (shown in **S2 Fig**) are surface exposed, which is consistent with the domain organizations in the full-length CSPG4 for the N-terminus of D1_401-560_ to connect to LGRs and the C-terminus to connect to D2 (**Fig 1A**) [25], while other parts of D1_401-560_ can still bind TcdB. D1 of CSPG4 was proposed to form disulfide bonds to stabilize the tertiary structure [26]. Accordingly, we observed one disulfide bond in our structure of D1_401-560_, formed by Cys415 in the loop before α1 and Cys533 on α5 (**S2 Fig**). CSPG4 is a heavily glycosylated protein. Asn427 in CSPG4 is predicted [27] to be N-linked glycosylated, following a sequence pattern of N-X-S/T **(S3 Fig)**. While the composition of the glycan is yet to be determined, in our cryo-EM density of D1_401-560_, we do observe a large density extending from the side chain of Asn427, which should correspond to the linked glycans (**S3 Fig).** However, this glycan does not interfere with TcdB binding, as the location of Asn427 is away from the TcdB binding sites.

### Detailed interactions between TcdB and CSPG4

**Fig 2:**
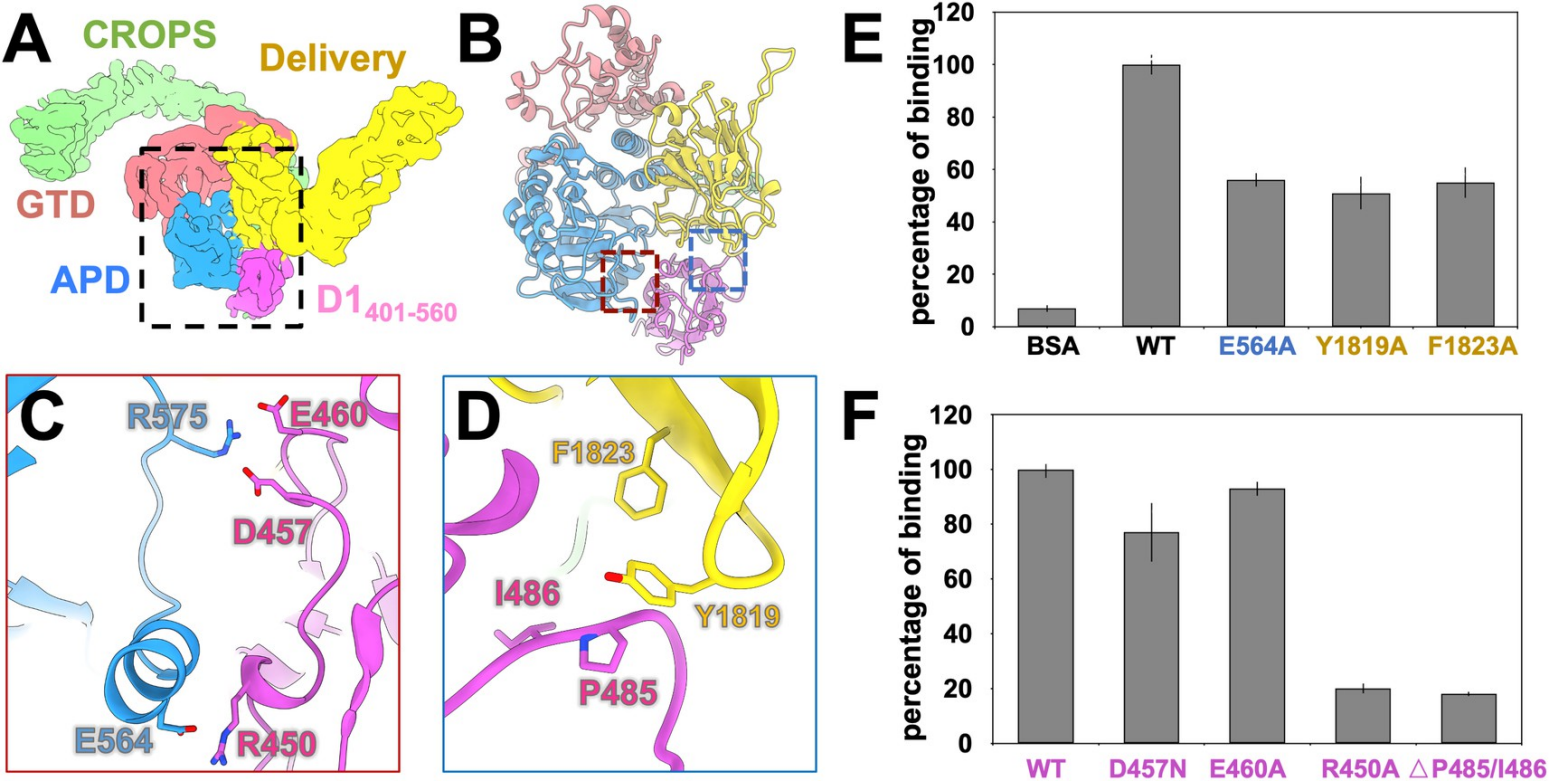
Detailed interactions at the TcdB-D1_401-560_ interface. (A) The density map of the full-length TcdB and D1_401-560_ complex, with each domain colored based on the scheme in **Fig 1**. The dashed black box indicates the region of focused refinement and contains the interfaces between TcdB and D1_401-560_. (B) The model of TcdB-D1_401-560_ complex for the density in dashed black box in Panel A. The dashed red and blue boxes indicate the positions of the 2 binding interfaces. (C) The interaction between TcdB APD and D1_401-560_ with the interacting residues labeled. (D) The interaction between the TcdB delivery domain and D1_401-560_ with the interacting residues labeled. Blue labels the mutation in the APD; yellow labels the mutations in the delivery domain. (E) ELISA of TcdB mutants to WT D1_401-560_. The mutations are labeled in the X-axis and the percentages of binding relative to WT D1_401-560_ are labeled on the Y-axis (raw data in S1 Data). (F) ELISA of WT TcdB to D1_401-560_ mutants. The mutations are labeled on the X-axis and the percentages of binding relative to WT TcdB are labeled on the Y-axis (raw data in S2 Data). Error bars in Panels E and F represent standard deviations (*n =* 4 and *n* = 2, respectively). APD, autoprocessing domain; CROPS, combined repetitive oligopeptides; GTD, glucosyltransferase domain; TcdB, Toxin B; WT, wild-type.

The 3.4-Å resolution cryo-EM density at the central part of the TcdB-D1_401-560_ complex (black dashed box in **Fig 2A**) allowed us to build an atomic model, which revealed 2 binding interfaces (labeled by red and blue dashed boxes in **Fig 2B**). One interface, between D1_401-560_ of CSPG4 and the APD of TcdB, is stabilized by a network of salt bridges (**Fig 2C**), formed between Arg575 (of the APD) and Asp457/Glu460 (in the loop connecting α2–3 in D1_401-560_), as well as Glu564 (of the APD) and Arg450 (in α2 of D1_401-560_). The other interface, located between D1_401-560_ and the delivery domain of TcdB, is stabilized by large aromatic residues from the D97 region at the C-terminus of the delivery domain and a loop between β3–4 of D1_401-560_ (**Fig 2D**). More specifically, Tyr1819 of the delivery domain interacts with Pro485 through hydrophobic effects and CH/π interactions [28,29]. Other hydrophobic residues, including Phe1823 in the delivery domain and Ile486 in D1_401-560_, were also identified at this interface to form hydrophobic interactions.

To further test the functions of these identified residues at the binding interfaces, we performed targeted mutagenesis on both TcdB and D1_401-560_. Mutants of TcdB and D1_401-560_ were cloned and expressed in BL21(DE3) and 293F cells, respectively **(S1 and S2 Tables)**. Of all the mutants we cloned, 7 of them were successfully expressed, purified, and characterized to be structurally stable (**S4 Fig and S6 and S7 Datas**). These mutants were then tested with ELISA to see if there is a change in binding (**Fig 2E and 2F**). Mutating Glu564 of TcdB or Arg450 of CSPG4 to an alanine significantly reduced the binding, due to the disruption of the salt bridge between them. Mutating either Asp457 or Glu460 of CSPG4 to an alanine only slightly reduced the binding. This is probably due to the fact that a single mutation on one of the 2 negatively charged residues is not sufficient to completely disrupt the electrostatic interactions to the opposite positively charged Arg575 on TcdB. All the mutants on Arg575 of TcdB failed to express soluble proteins. Fortunately, TcdB subtype 3 has a glycine at residue 575 instead of an arginine (**S3 Fig**) and does not bind well to CSPG4 [30], which can be explained by the disruption of the electrostatic interaction with the opposite Asp457 and Glu460. For the interactions at the interface between the delivery domain of TcdB and CSPG4, alanine mutations of either Tyr1819 or Phe1823 reduce the binding about 1-fold (**Fig 2E and S1 Data**), while the deletion of both Pro485 and Ile486 on the opposite side compromises the binding significantly (**Fig 2F and S2 Data**).

### Change in receptor binding upon acidification

**Fig 3:**
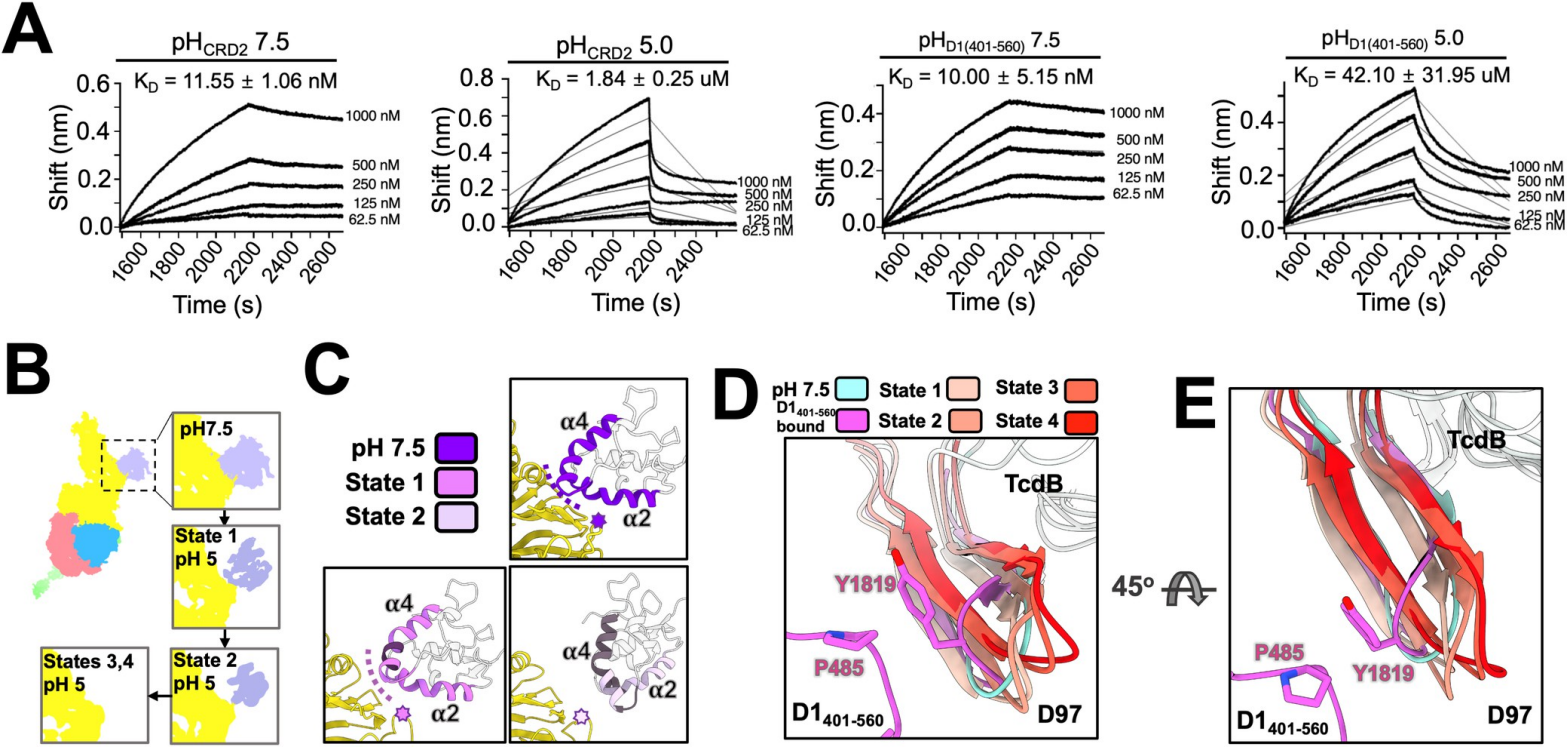
Interactions between TcdB and its receptors under different pHs. (A) Binding kinetics between TcdB and receptors are measured by BLI. The calculated binding affinities of TcdB to the 2 receptors, under pH 7.5 or pH 5, are labeled above each diagram. This measurement was calculated based on 2 biological replicates with standard deviations listed for each sample (raw data in S3 Data). (B) The structure comparisons of the TcdB-CRD2 complex at pH 7.5 and the 2 states at pH 5. (C) The helices (α2 and α4) of CRD2 that interact with TcdB are colored for each state. Dashed curves label the CRD-binding groove. Stars label the CRD-binding loop. Dark gray color labels regions in α-helices 2 and 4 that lack the cryo-EM density. The buried surface areas, calculated based on the model for the complex of TcdB and CRD2 under pH 7.5, state 1 and state 2 are around 700, 150, and 80 Å^2^, respectively. (D) The movement of the β-sheet (residues 1,812–1,827) in the D97 region are shown and color coded for each state. Tyr1819 of TcdB and its interaction partner Pro485 of CSPG4 are shown. (E) The same region rotated 90° from Panel D. BLI, bio-layer interferometry; CRD, cysteine-rich domain; CRD2, cysteine-rich domain of frizzled-2; TcdB, Toxin B.

At cytosolic pH (pH 7.5), both CRD2 (CRD of FZD2) and D1_401-560_ have high affinities for TcdB, with a K_D_ of 19 nM and 29 nM, respectively [11,12]. During endocytosis, acidification of the late endosome (pH 5) is proposed to cause a conformational change of TcdB, preparing it for the penetration of the endosomal membrane required for the delivery of effector domains into the cytosol [23,31–33]. However, it is not clear how acidic pH will affect the binding between TcdB and the host receptors. To answer this question, we assessed the binding of TcdB to CRD2 and D1_401-560_ using bio-layer interferometry (BLI) at cytosolic (pH 7.5) and acidic (pH 5) pHs (**Fig 3A and S3 Data and S5 Fig**). Consistent with previous publications [11,12], K_D_ values for TcdB with CRD2 and D1_401-560_ are approximately 12 nM and 10 nM, respectively, at pH 7.5. The binding for TcdB with both receptors weakened upon acidification. Particularly, the K_D_ for TcdB with CRD2 increases to approximately 1 μM, while the K_D_ for TcdB with D1_401-560_ drastically increases to tens of μM (**Fig 3A and S3 Data**), which is consistent with the ELISA analysis performed under pH 7.5 or pH 5 (**S6 Fig and S8 Data**).

To obtain a structural explanation for the retained binding of CRD2 at acidic pH, we performed single-particle cryo-EM on the complex of full-length TcdB with the CRD2 at both pH 7.5 and pH 5 (**Fig 3B**). At pH 7.5, a consensus map of TcdB-CRD2 complex can be obtained at 5.1 Å resolution (**S7 Fig and S9 Data**). The CRD2 consists of 2 β-strands and 5 α-helices (**S8 Fig**). Our structure reveals that α-helices 2 and 4 of the CRD2 insert into a “CRD-binding groove” (labeled by purple dashed curves in **Fig 3C**), formed by an α-helix (residues 1,432 to 1,439) and a β-sheet (residues 1,492 to 1,510) in the delivery domain of TcdB. In addition, α-helix 2 of the CRD2 interacts with a “CRD-binding loop” (residues 1,596 to 1,600, labeled by purple stars in **Fig 3C**) in the delivery domain. Such a binding mode is consistent with the previous crystal structure of the complex between CRD2 and the fragment of the TcdB delivery domain [14].

Flexibility of TcdB-CRD2 complex at pH 5 was observed using 3D variability analysis (**S1 Movie and S9 Fig**). At pH 5, we observed multiple states of the TcdB-CRD2 complex (**S10 Fig and S10 Data**), leading to 4 reconstructions (States 1 to 4) resolved at 4.1 Å, 5.1 Å, 4.6 Å, and 5.9 Å, respectively. Interestingly, for the 4 states at pH 5, different CRD2 binding modes were observed. About 30% of the particles at pH 5 (State 1) have an almost complete density for the CRD of FZD2 (**Fig 3B**). In this state, the CRD2 binds to TcdB in a similar mode as at pH 7.5 (**Fig 3B and 3C**), except for the α-helix 4, which has the middle of its density missing, and α-helix 2, which distorts and shifts away from the center of CRD2 (**S8 Fig**). Strikingly, approximately 23% of the particles of TcdB still bind to the CRD2, but with a much weaker CRD2 density (State 2), which only accounts for 54% of its complete structure (**Fig 3B**). Closer inspection of the model for State 2 reveals that part of the density for α-helices 2–4 is missing (**S8 Fig**). Instead of inserting into the CRD-binding groove in the delivery domain, the N-terminal region of the α-helix 4 of CRD2 shifts its interaction to the CRD-binding loop (residues 1,596 to 1,600) (**Fig 3C**). Lowering the pH may have partially unfolded the CRD2, leading to States 1 and 2 as intermediate states preceding the complete dissociation of CRD2 from TcdB (**S8 Fig**). Another 2 states (States 3 and 4) are free of the CRD2 and account for approximately 47% of the particles. The differences between States 3 and 4 are mostly in the CROPS domain (**S11 Fig**). We also notice that the D97 region of the delivery domain becomes more flexible upon acidification (**S2 and S3 Movies**), which explains this larger variation in the connected CROPS domain at pH 5.

Of note, for all the structures of the TcdB-CRD2 complex we solved, we did not observe the density for the palmitoleic acid (PAM) in CRD2, which was proposed to serve as a coreceptor for TcdB binding [14]. The associated PAM may have been lost in the purchased CRD2 during the lyophilizing of the proteins. Nevertheless, it is intuitive that the additional PAM should enhance the hydrophobic interaction, which may stabilize the CRD2 upon acidification and lead to more CRD-bound TcdB at pH 5.

For CSPG4, there is a dramatic decrease in binding to TcdB at pH 5 (**Fig 3A**). This can be explained by the fact that the interaction between D1_401-560_ of CSPG4 and the APD of TcdB is maintained by salt bridges (**Fig 2C**), which will be compromised at low pH [34]. Upon acidification, we also observed that the β-strand in the D97 region, which contains the Tyr1819 that forms aromatic/proline interaction with D1_401-560_, becomes flexible and moves away from the D1_401-560_ binding site (**Fig 3D and 3E and S12**). Such a movement may cause the dissociation of the Tyr1819 of TcdB from the Pro485 in D1_401-560_, therefore further releasing D1_401-560_ from TcdB. Acidification may also cause partial unfolding of CSPG4, though we cannot visualize it through a cryo-EM structure of the TcdB-D1_401-560_ complex under pH 5 due to its low-binding affinity to TcdB under acidic pH.

## Discussions

**Fig 4:**
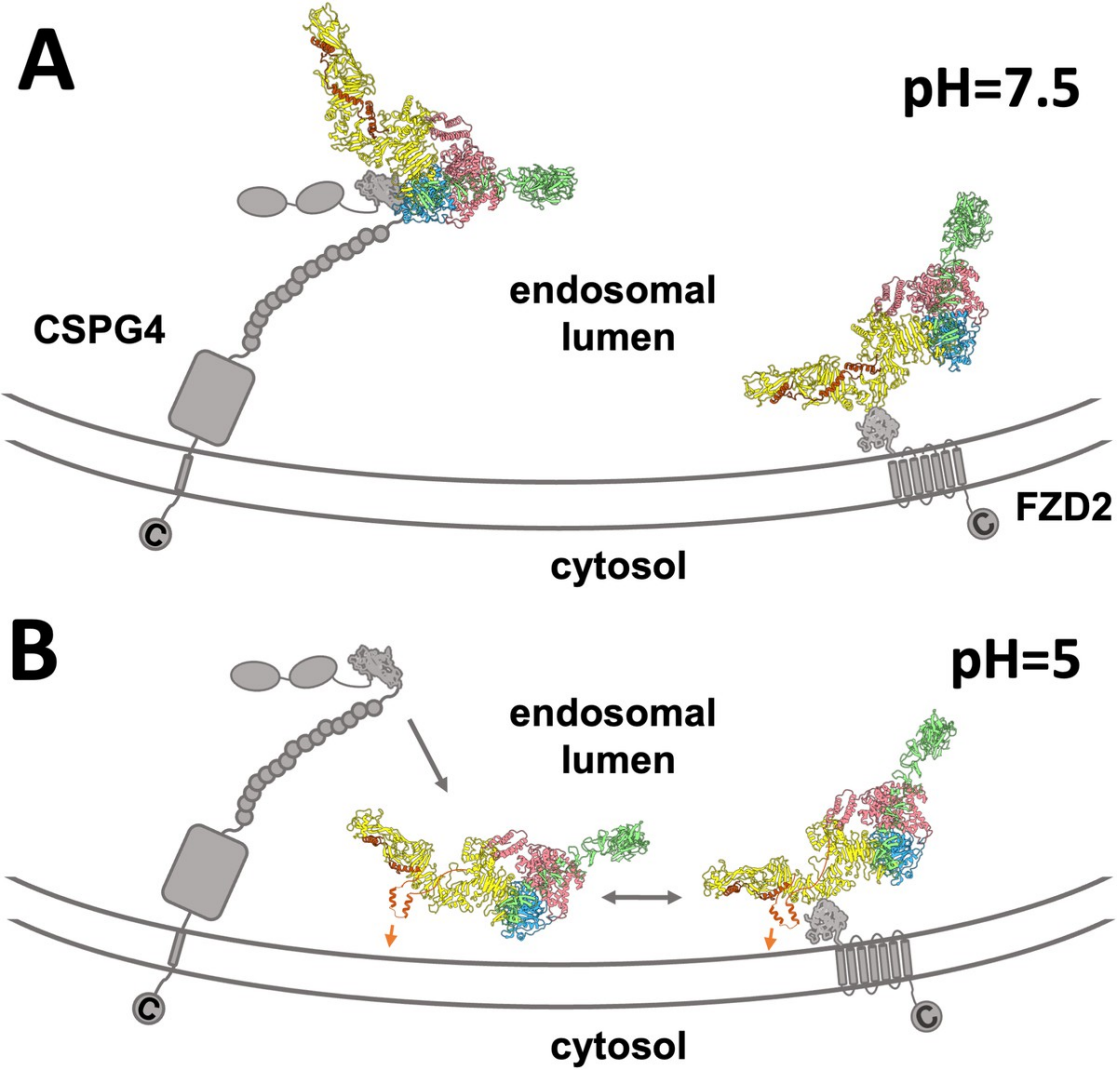
The proposed model of the recognition of CSPG4 and FZD2 by TcdB and its dissociation upon endosomal acidification. (A) TcdB (shown as a ribbon model) binds to the receptors (gray cartoon), CSPG4 and FZD2, before endosomal acidification. The proposed pore-forming helices of TcdB are shown as orange ribbon models. (B) TcdB dissociates from the receptors and the pore-forming helices rearrange. Gray arrows indicate the dissociation of TcdB. Orange arrows suggest the insertion of pore-forming helices into the endosomal membrane. CSPG4, chondroitin sulfate proteoglycan 4; FZD2, frizzled-2; TcdB, Toxin B.

In this study, we have resolved the structure of the TcdB-binding domain D1_401-560_ of CSPG4 and revealed its molecular interfaces to TcdB. Upon acidification, TcdB is drastically released from CSPG4, due to the compromised electrostatic interactions at acidic pH and the destabilization of the D97 region in the TcdB delivery domain. In contrast, FZD2 still moderately binds to TcdB at pH 5. Such a difference in dissociation for the 2 receptors is compatible with the size difference of their extracellular domains (**Fig 4**). To translocate the effector domains of TcdB into the cytosol, several pore-forming helices, which are embedded in the center of the delivery domain of TcdB, have to get close to the endosomal membrane to form pores. The extracellular domain of CSPG4 is bulky (approximately 220 kDa for protein and approximately 200 kDa for glycans) and binds TcdB with D1_401-560_, which can be over 400 Å away from the endosomal membrane. If TcdB were still associated with D1_401-560_ upon acidification, it would be unable to access the endosomal membrane for pore formation. On the other hand, frizzled proteins, such as FZD2, have a much smaller extracellular domain (approximately 25 kDa) and bind TcdB in the middle of its delivery domain, close to these pore-forming helices. The partial dissociation between the CRD domain of FZD2 and TcdB may keep the delivery domain proximal to the endosomal membrane, facilitating the pore formation. Indeed, during acidification, we observed rearrangements of the pore-forming helices in our cryo-EM structures, which may prime it for the insertion into the endosomal membrane (**S11 Fig**). Such a rearrangement of the delivery domain is consistent with a previously reported crystal structure of TcdB at acidic pH, in which some of the pore-forming helices are disordered [9]. Since upon acidification, FZD2 has a higher affinity than CSPG4 in binding TcdB, we cannot rule out the possibility that dissociated TcdB from the bulky extracellular domain of CSPG4 could reassociate with FZD2, allowing the 2 receptors to work cooperatively for the cell entry of TcdB. It has been observed that TcdB can intoxicate cells that only express CSPG4 [18]. One explanation is that after being recruited by the CSPG4 and dissociated into the endosome upon acidification, the confined space within the late endosome will allow TcdB to frequently diffuse close to the endosomal membrane to insert the pore forming helices without the help from another receptor, such as the FZD2.

The movement of the TcdB-CRD2 complex at pH 5 is continuous rather than discrete based on the 3D variability analysis (**S1 Movie**). Thus, the 4 states of the TcdB-CRD2 complex at pH 5 were the representative states that were clustered out of all the conformations. It was shown before that TcdB takes more conformations at acidic pH compared to neutral pH due to increased flexibility of the CROPS domain [35]. The mechanistic origins for the pH-induced flexibility of the CROPS domain are not clear. However, based on our structures (shown in **Fig 3D** and **3E**), the D97 region, which is upstream of the CROPS domain, exhibits conformational changes and may be involved in the coordination of the observed flexibility.

One strategy in treating CDI is to neutralize the *C*. *difficile* toxins instead of killing the bacteria. Such a strategy targets the virulence factors, reducing the pathogenicity of the bacteria and minimizing selective pressure on *C*. *difficile* to develop drug resistance. Our structures of TcdB, complexed with either D1_401-560_ of CSPG4 or CRD of FZD2 under neutral and acidic pH, provide a basis for developing therapeutics against CDI. For example, one could develop antibodies or antibody-like proteins, which can bind to TcdB and shield its binding sites for host receptors, thus preventing its cell entry. One previously reported antibody-like protein, a dual modular DARPin DLD-4, shows promise in neutralizing the toxicity of TcdB [36,37]. By comparing the structure of the TcdB/DLD-4 complex with the structures reported in this paper, we see that the 2 modules of DLD-4 block the binding sites for FZD2 and CSPG4 on TcdB (**S14 Fig**).

The effector domain of TcdB glucosylates small GTPases to disrupt the assembly of cytoskeletons inside the cell, causing cell rounding, which eventually leads to cell death [38]. CSPG4, the receptor for TcdB, is abundantly expressed in cancers [26,39] including over 70% of melanomas. CSPG4 is also expressed in the triple-negative breast cancer, which is insensitive to available targeted therapies. Results from our study may provide a basis for developing new toxin-based therapeutics to selectively target those cancer cells that express CSPG4. The idea of using bacterial toxins in cancer treatment is not unfounded, as TcdB has been used to induce antitumor immunity [40]. To develop such a toxin-based anticancer therapeutic, more validation through cell biology and immunology is needed.

A recent published paper [22] reported a cryo-EM structure of a TcdB fragment in complex with CSPG4 D1 fragment. Our observed interactions between the full-length TcdB and CSPG4 D1 fragment are consistent with their results. For example, the identification of Glu564 of APD was involved in charge–charge interaction with Arg450 from CSPG4; Phe1823 of the delivery domain is involved in the hydrophobic interaction with Pro485 from CSPG4. In our paper, we also performed mutagenesis on the CSPG4 to provide a more complete view of the interactions. In addition, we present multiple structures of receptor-bound TcdB under different pH to reveal the impact of acidification on the receptor binding process. The use of a full-length TcdB also allows us to measure the conformational flexibility of different domains, particularly the CROPS domain, which is missing in the other study.

## Methods

### D1_401-560_ expression and purification

The cDNA sequence of the CSPG4 N-terminal secretion signal and D1_401-560_ is synthesized and cloned into pEGFP-N1 plasmid with a green fluorescent protein (GFP) tag attached to the C terminus. FreeStyle 293-F cells were seeded into fresh FreeStyle 293 expression media with a final density of 1.0 × 10^6^ cells ml^−1^ and incubated at 37°C, 8% CO_2_, 130 rpm. After 24 hours, 2 mg of the pEGFP-N1-D1_401-560_ plasmids and 4 mg of the linear PEI25000 (Polysciences) were mixed into 100 ml 1 × PBS and incubated at room temperature for 20 minutes. Then, the mixture was added into 2L FreeStyle 293-F cells. After 6 days, the cells were pelleted at 3,000 rpm for 5 minutes. One protease cocktail inhibitor tablet (Roche) and subsequently 2 mL streptavidin agarose beads (EMD Millipore) were added into the supernatant. The streptavidin agarose beads have been coupled with biotin-labeled Avi-SUMO-GFP nanobody. After shaking at 4°C overnight, the beads were pelleted and washed with a prechilled PBS buffer. Finally, the beads were resuspended in a 2-mL washing buffer and SUMO protease was added to cleave the target protein from the beads. To fully elute the protein, the beads were then rewashed using another 2 mL of the washing buffer. The eluted protein was further purified using a superdex 200 increase 10/300 GL column (GE Healthcare). The D1_401-560_ mutants were expressed in 450 mL FreeStyle 293-F cells and purified through the same approach as the WT D1_401-560_.

### Plasmid reconstructions of TcdB and CSPG4 mutants

Primers are designed based on the sequence of pHis22-TcdB (sequence from VPI 10463 strain) from residue 550 to residue 1,902. The PCR product was pasted on pET28a plasmid with a His-sumo tag on its 3′ end. The plasmid that contained the correct sequence of TcdB truncation was saved as a template for the following site-directed mutagenesis for TcdB mutants. Following the instruction of the Q5 site-directed mutagenesis kit (New England Biolabs), primers containing the mutated base pairs (**S1 Table**) were used for plasmid reconstructions. The mutations on D1_401-560_ were conducted similarly to the description above. The primer designs for the mutants were listed in **S2 Table.**

### TcdB expression and purification

pHis22-TcdB was transformed into *Bacillus megaterium* cells, and the recombinant TcdB with C-terminal 6XHis-tag was purified via Ni-NTA affinity column as previously described [41]. The bound protein was eluted using high-salt PBS (20 mM NaH_2_PO_4_, 20 mM Na_2_HPO_4_, 300 mM NaCl at pH 7.5) containing 250 mM imidazole. The eluted protein was then loaded onto a superdex 200 increase 10/300 GL column (GE Healthcare), and all the fractions are confirmed using SDS-PAGE. The verified eluted TcdB is concentrated using concentrators (Amicon Ultra-15 Centrifugal Filter Units, Millipore). TcdB mutants were expressed in *E*. *coli* BL21(DE3) with an overnight expression at 18°C at 200 rpm and purified similarly as described above.

### Cryo-EM sample preparation of TcdB-D1_401-560_ complex and TcdB-CRD2 complex at pH 7.5

TcdB and FZD2-CRD-Fc (R&D Systems) or D1_401-560_ were mixed at 1:1 molar ratio (with the final concentration of the complex at 800 nM) and incubated in PBS buffer at pH 7.5 for 30 minutes at room temperature, respectively; 3 μL of the complex (with a concentration of 2 mg/mL) was applied to C-flat 2/1 holey carbon film 300 mesh grids (Electron Microscopy Sciences, PA) at 20°C with 100% relative humidity and vitrified using a Vitrobot (Mark III, FEI Company, the Netherlands). The grids were stored in liquid nitrogen till usage. To avoid the preferred orientation in the TcdB-D1_401-560_ sample, 0.01% DDM was added.

### Cryo-EM sample preparation of TcdB-CRD2 complex at pH 5

The purified TcdB (0.3 mg/mL) was preincubated with FZD2-CRD-Fc (R&D Systems, Minneapolis, MN) in a 50-mM PBS buffer at 1:1 ratio under room temperature and pH 7.5 prior to the pH change. The protein complex was mixed with a 50-mM citric acid buffer (adjusted to pH 5) at ratio 7:3 before applying on the grid. Around 3 μL of the pH changed sample was applied to the C-flat 2/1 holey carbon film 300 mesh grids (Electron Microscopy Sciences, PA) and incubated for 45 seconds before freezing.

### Cryo-EM data collection for the TcdB-D1_401-560_ and TcdB-CRD2 complexes

Both the TcdB-D1_401-560_ and TcdB-CRD2 complexes were imaged under a Titan Krios G3 transmission electron microscope (Thermo Fisher Scientific) operated at 300 kV. The microscope is equipped with a Gatan K2 summit direct detection camera (Gatan, Pleasanton, CA). A total of 17,206 micrographs and 5,478 micrographs were collected using electron-counting mode at a pixel size of 1.06 Å for TcdB-D1_401-560_ complex and TcdB-CRD2, respectively. The beam intensity was adjusted to 7e^−^/Å^2^/s on the camera. For each micrograph, a 33-frame movie stack was collected at 0.2 seconds per frame for a total exposure time of 6.6 seconds. The grid of TcdB-CRD2 at pH 5 was imaged under the same microscope, but with a Gatan K3 summit direct detection camera (Gatan, Pleasanton, CA). A total of 11,000 micrographs were collected using electron-counting mode at a pixel size of 0.732 Å. Another 1,543 micrographs of the TcdB-CRD2 complex were collected under the JEOL JEM3200FSC electron microscope (JEOL, Japan), operated at 300 kV, and equipped with a Gatan K2 summit direct detection camera. The beam intensity on JEM3200FSC was adjusted to 5 e^−^/Å^2^/s on the camera. A 33-frame movie stack was collected for each micrograph, with 0.2 seconds per frame, for a total exposure time of 6.6 seconds. An in-column energy filter was used with a slit width of 29 eV.

### Data processing for TcdB-D1_401-560_ complex at pH 7.5 and TcdB-CRD2 at pH 5

The collected micrographs for the TcdB-D1_401-560_ complex were motion-corrected and dose-weighted using MotionCorr2 [42]. A total of 16,135 micrographs with strong power spectra were selected for further processing. Contrast transfer functions (CTFs) of the micrographs and the following processing were all done in cryoSPARC [43]. The blob-picked particles were used to generate the initial 2D classification result, providing a template for the particle picking tool. The picked particles were scaled to a pixel size of 4.24 Å before the final 2D classification. Ab initio reconstruction was performed, and its output was used for subsequent heterogenous refinement. A total of 470,301 clean particles were selected to generate the full map, yielding a 4.8-Å resolution density map. A local refinement was then performed after subtracting the signal from the tip of the delivery domain and the CROPS domain, yielding a density map at the central region of the complex at 3.4 Å resolution (**S13 Fig**). The overall resolution was assessed using the gold-standard criterion of Fourier shell correlation (FSC), with a cutoff at 0.143, between 2 half maps from 2 independent half-sets of data. Local resolutions were estimated using Resmap [44].

Similar approach was used to generate a consensus map of the TcdB-CRD2 complex at pH 5 using 333,554 particles (**S9 Fig**). Then, we performed a 3D variability analysis on the particles since flexibility was observed in the consensus map. Three principal components were requested during analysis, and relative movements were observed on the delivery and CROPS domains (**S1 Movie**). After this, we did an ab initio reconstruction on the selected clean particles, defining the class similarity to 1. Since 4 clusters were identified in the variability analysis, 4 classes of the reconstruction were requested during the heterogeneous refinement, followed by homogenous refinement on each of the classes. These 4 classes were the 4 states for comparison (**S10 Fig**).

### Data processing for TcdB-CRD2 complex at pH 7.5

A total of 6,368 motion-corrected micrographs with strong power spectra were selected for further processing. CTFs of the micrographs were estimated using Gctf [45]. Particles were picked using Gautomatch with 2D templates derived from previously published density maps [36]. These particles were scaled to a pixel size of 4.24 Å using RELION-3.0 [46]. The automatically picked particles were then screened for high-contrast particles through 4 rounds of the reference-free 2D classification. A total of 146,129 clean particles were selected and combined for 3D classification. Then 81,240 clean particles from 3D classification were used for 3D refinement. The overall resolution of the cryo-EM map for the TcdB-CRD2 complex at pH 7.5 is 5.1 Å, estimated as described above.

### Model building of the TcdB-D1_401-560_ complex

The 3.4-Å density map was used for the TcdB-D1_401-560_ model building. The initial model of D1_401-560_ was generated by the Robetta server [47]. The generated initial model was carefully fitted in the density map to trace the backbone. The positions of glycosylation for Asn427 and the C-terminal GFP tag, which were shown in the density map, served as 2 anchors for the backbone. This backbone model was then refined in RosettaCM [48], producing 1,000 atomic models for D1_401-560_. Six models were selected based on the lowest energy score as well as the best fitting to the EM density (**S3 Fig and S5 Data**). One best model was then iteratively refined in ISOLDE [49] and Phenix [50]. The model of D1_401-560_ was then combined with the TcdB homolog model [51] and used for another round of ISOLDE refinement as well as the Phenix real-space refinement.

### Model building of TcdB-CRD2 complex at pH 7.5 and at pH 5

The homolog model of TcdB and CRD2 were combined and docked into the EM density map in UCSF Chimera [52] and refined into the cryo-EM density map using Molecular Dynamics Flexible Fitting [53] to generate the complex structure of TcdB and FZD2-CRD, which was further refined using Phenix [49].

### ELISA of wild-type TcdB and wild-type or mutant D1_401-560_ at pH 7.5

Biotinylated TcdB was generated using the EZ-Link Sulfo-NHS-LC-Biotin kit. MaxiSorp immuno plates (Nunc) were coated with 16 nM TcdB overnight at 4°C. The next day, the wells were washed and blocked with PBSTB buffer (PBS containing 0.1% Tween-20 and 2% BSA) before being incubated with 50 nM wild-type D1_401-560_ or mutant D1_401-560_. After incubation, wells were washed 4 times with PBST. Bound D1_401-560_-EC-GFP was detected using a rabbit anti-GFP antibody (0.05 μg/mL, Proteintech, catalog #50430-2-AP) and an HRP-conjugated anti-rabbit antibody (0.8 μg/mL, Santa Cruz Biotechnology, catalog #SC-2004). The color development agent was 3,3′,5,5′-tetramethylbenzidine (TMB).

### ELISA of TcdB mutants and WT D1_401-560_ at pH 7.5

A similar approach was used as described above. MaxiSorp immuno plates (Nunc) were coated with 20 nM WT D1_401-560_ overnight at 4°C. The next day, the wells were washed and blocked with PBSTB buffer (PBS containing 0.1% Tween-20 and 2% BSA) before being incubated with 16 nM truncated wild-type TcdB or mutated TcdB (containing a His-tag at the C-terminus). After incubation, wells were washed 4 times with PBST. Bound TcdB was detected using a mouse anti-His antibody (0.5 μg/mL, R&D Systems, catalog #MAB050-SP) and an HRP-conjugated anti-mouse antibody (0.8 μg/mL, Jackson ImmunoResearch, catalog #: 115-035-146).

### ELISA of wild-type TcdB with wild-type CRD2 or D1_401-560_ at different pHs

Approximately 10 nM TcdB-biotin was combined with 0.5 nM D1_401-560_ or 0.25 nM FZD2-CRD-Fc at pH 7.5 and incubated at room temperature for 1 hour. The pH of each mixture was shifted to pH 5 or kept at pH 7.5 and incubated at room temperature for 30 minutes. Mixtures were then added to streptavidin-coated ELISA wells to capture TcdB-biotin complexes and incubated for 1 hour. Wells were first washed twice with a pH 7.5 or pH 5 buffer, respectively, and then all the wells were rewashed 4 times with pH 7.5 buffer. After washing, TcdB-bound D1_401-560_ was detected with an anti-GFP antibody, and TcdB-bound FZD2-CRD-Fc was detected with an anti-Fc antibody. The amount of TcdB-biotin captured was quantified using streptavidin-HRP. The blocking buffer used was PBSTB buffer (PBS containing 0.1% Tween-20 and 2% BSA).

### Bio-layer interferometry (BLI) assays

BLI experiments were performed using the Octet Red 96 instrument (FortéBio). His-tagged full-length TcdB and receptor proteins (CRD2, D1_401-560_) were diluted in an assay buffer containing 10X Octet Kinetics Buffer (Sartorius, MN) diluted to 1X with PBS. The final concentration of TcdB was 20 μg/ml while receptor proteins were serially diluted within a range of 1 μM to 62.5 nM. TcdB was first bound to the Ni-NTA biosensors, followed by a washing step with assay buffer and an association step of the receptor protein to the sensor-bound TcdB. Then, all biosensors were washed with the assay buffer, which was either pH 7.5 or 5.0 for the dissociation. The assay was performed in solid black 96-well plates (Greiner Bio-One), using agitation set at 1,000 rpm, and temperature set at 25°C. Experiments were done with duplicates.

### Binding affinity (K_d_) kinetics analysis

Reference values from control wells, which have no receptor proteins, were subtracted and all data in trace by value were aligned using Octet Data Analysis 11.1.2 software. After this processing, data were further curve-fitted with association-then-dissociation model using GraphPad Prism v.9.3.1 (GraphPad Software).

### Thermal shift assay

The thermal stability of D1_401-560_ mutants was measured using label-free, native differential scanning fluorimetry (nanoDSF; Prometheus NT.48, NanoTemper). Each mutant protein was diluted with 1X PBS until the final concentration became 0.15 mg/ml. The tryptophan residues of the proteins were excited at 280 nm, and the fluorescence intensity was recorded at 330 and 350 nm. The temperature of the measurement compartment increased from 25 to 85°C at a rate of 1°C/min. Two-state Tm_1_s were obtained using PR.ThermControl Software (NanoTemper).

### Circular dichroism spectra of TcdB mutants

Approximately 0.1 mg/mL purified TcdB mutants in phosphate buffer (50 mM phosphate buffer (pH 7.5) 150 mM NaCl) were used. Circular dichroism spectra (Chirascan, Applied Photophysics) were recorded from 190 to 280 nm at constant temperature in a 0.1-cm-pathlength cell. The spectral bandwidth was 1 nm, the step size was 1 nm, and the averaging time was 1.5 seconds.

## Supporting information

S1 Fig The map quality validation of the TcdB-D1_401-560_ complex. **(**A) Representative micrograph and 2D classifications of the TcdB-D1_401-560_ complex. (B) The orientation distribution (top) and local resolution (bottom) for the reconstruction of the central region of the TcdB-D1_401-560_ complex. (C) The representative densities show bulky side chains. (D) FSCs of the cryo-EM reconstructions (S4 Data). (E) The local resolution for the reconstruction of the complete TcdB-D1_401-560_ complex. FSC, Fourier shell correlation; TcdB, Toxin B.
(PNG)

S2 Fig The structure of D1_401-560_. (A) The topology of D1_401-560_ with α-helices and β-strands shown as blue cylinders and red arrows, respectively. The N- and C-termini are labeled by circles. The blue stars and the yellow star highlight the binding regions for APD and the delivery domain of TcdB, respectively. (B) The atomic model of D1_401-560_. A disulfide bond is present and residue N427 for glycosylation is labeled. (C) The model of D1_401-560_ rotated 90° from the view in Panel B. The dimensions of D1_401-560_ are labeled. APD, autoprocessing domain; TcdB, Toxin B.
(PNG)

S3 Fig Model building and validation for D1_401-560_. (A) The diagram shows the overall energy and the EM density fit score for 1,000 models of D1_401-560_ generated by RosettaCM [48]. The 6 chosen models were labeled in red circles (S5 Data). (B) The glycosylation site was predicted through the webserver NetNGlyc-1.0 [27] and highlighted in red (top). The density of the potential glycan on Asn427 of D1_401-560_ is shown (bottom). (C) The primary sequence alignment among TcdB1, TcdB2, and TcdB3 from residue 560 to residue 590. The sequence differences were highlighted. The residue 575 of the 3 TcdB subtypes were boxed. (D) Extra density for the GFP tag indicates the position of the D1_401-560_ C-terminus. CSPG4, chondroitin sulfate proteoglycan 4; GFP, green fluorescent protein; TcdB, Toxin B.
(PNG)

S4 Fig TcdB and D1_401-560_ mutants remain similar structures as wild types. (A) Representative circular dichroism spectroscopy on TcdB mutants. Measurements were repeated twice and produced similar results (S6 Data). (B) The thermal stability of mutant D1_401-560_ of CSPG4 proteins were measured using label-free, native differential scanning fluorimetry (nanoDSF; Prometheus NT.48, NanoTemper). The data are presented as means ± SD (*n =* 3). D1_401-560_ variants showed Tm_1_ values comparable to the wild-type D1_401-560_ protein, which implies the correct protein folding (S7 Data). CSPG4, chondroitin sulfate proteoglycan 4; TcdB, Toxin B; WT, wild type.
(PNG)

S5 Fig Representative raw data from the BLI assay. Representative binding curves of 4 different conditions after TcdB, as a ligand, are immobilized on Ni-NTA biosensors. The concentrations of the receptor proteins (FZD2-CRD, D1_401-560_) are labeled in each panel. The reference wells, which assay buffer was added instead of receptor proteins in association step, are depicted in purple. (A) pH 7.5 dissociation after CRD2 binding. (B) pH 5.0 dissociation after CRD2 binding. (C) pH 7.5 dissociation after D1_401-560_ binding. (D) pH 5.0 dissociation after D1_401-560_ binding. BLI, bio-layer interferometry; CRD2, cysteine-rich domain of frizzled-2; FZD2, frizzled-2; TcdB, Toxin B.
(PNG)

S6 Fig ELISA assay on binding affinity of TcdB and its receptors under neutral pH and acidic pH. The signals of bound D1_401-560_/CRD2 at pH 7.5 were scaled to the same level and defined as 100% binding (S8 Data). Error bars represent standard deviations (*n* = 2). CRD2, cysteine-rich domain of frizzled-2; TcdB, Toxin B.
(PNG)

S7 Fig The density map of TcdB-CRD2 at pH 7.5. (A) The FSC of the cryo-EM reconstruction (S9 Data). (B) Two views of the TcdB-CRD2 density map at pH 7.5. Each TcdB domain was colored based on the scheme in **Fig 1**. The CRD of FZD2 (or CRD2) is colored purple. CRD, cysteine-rich domain; CRD2, cysteine-rich domain of frizzled-2; FSC, Fourier shell correlation; FZD2, frizzled-2; TcdB, Toxin B.
(PNG)

S8 Fig The rigid body docking of CRD2 into the cryo-EM maps. (A). The model of the CRD2 with secondary structures labeled. (B). The model of CRD2 (purple ribbons) from the structure of the TcdB-CRD2 complex, at pH 7.5, was docked into the density maps (transparent gray) in state 1 and state 2. The fitting of ɑ2, ɑ3, and ɑ4 in the densities for the pH 7.5 state, pH 5 State 1, and pH 5 State 2 are shown. The proposed unfolded regions were marked by black dashed circles. All the CRD2 densities were kept at the same surface area level for comparison (approximately 7,400 Å^2^). CRD2, cysteine-rich domain of frizzled-2; TcdB, Toxin B.
(PNG)

S9 Fig Data processing for the TcdB-CRD2 complex at pH 5. See Methods for details. In brief, 4,005,046 particles were autopicked from 11,134 motion corrected micrographs. The picked particles were further cleaned using 2D classification, ab initio reconstruction, and heterogeneous refinement. Only one class of heterogeneous refinement generated a good density map. A total of 333,554 particles from this class were used for consensus 3D refinement followed by 3D variability analysis. The variability analysis showed that the TcdB-CRD2 complex at pH 5 exhibited continuous conformational changes. Based on the principal components 1–3, we were able to distinguish the particles into roughly 4 conformations (top and bottom based on Components 1 and 2, left and right based on Components 2 and 3). Therefore, in the second round of heterogeneous refinement, we requested for 4 states of the TcdB-CRD2 complex at pH 5. These 4 classes were used as initial models for further refinement of each class. Local refinement was then applied by using a mask around the delivery domain or core region to obtain higher-resolution densities for model building. CRD2, cysteine-rich domain of frizzled-2; TcdB, Toxin B.
(PNG)

S10 Fig The density maps of TcdB-CRD2 at pH 5. (A) A representative micrograph and 2D classifications of the TcdB-CRD2 complex at pH 5. The projections with CRD2 density were highlighted with red boxes. The binding position was illustrated by red arrows. (B) FSCs of the 4 cryo-EM reconstructions of the TcdB-CRD2 complex at pH 5 (S10 Data). (C) Local resolutions (top) and orientation distributions (bottom) of 4 states of the TcdB-CRD2 at pH 5. All the maps were displayed at the same contour level (0.033) with cyan for high resolutions and maroon for low resolutions. CRD2, cysteine-rich domain of frizzled-2; FSC, Fourier shell correlation; TcdB, Toxin B.
(PNG)

S11 Fig The flexibility of TcdB at pH 5. (A) The scheme of domain movements between states at pH 5. TcdB domains are colored based on the scheme in **Fig 1**. (B) The rearrangement of the pore-forming helices from pH 7.5 (blue ribbon model) to pH 5 (ribbon models with different shades of red), as well as the crystal structure of TcdB at pH 5 (yellow: PDB: 6OQ5) [9]. At pH 5, helices consist of residues 1,039–1,050, and residues 1,054–1,065 become flexible and unresolved. The structures are aligned based on the delivery domain. As in our cryo-EM structures at pH 5, the X-ray structure also has helices 1,039–1,050 and 1,054–1,065 disordered. Interestingly, residues 1,049–1,054, which was originally a loop connecting 2 helices at pH 7, has changed into a short helical structure and flipped outside the core of the delivery domain, potentially for the membrane insertion. FZD2, frizzled-2; TcdB, Toxin B.
(PNG)

S12 Fig Map fitting of the model for the Tyr1819-containing β-sheet (yellow, residues 1,812–1,827) of TcdB. TcdB, Toxin B. (PNG)

S13 Fig Flowchart of the TcdB-D1_401-560_ data processing. The details are provided in Methods.
(PNG)

S14 Fig DLD-4 DARPin competes with TcdB receptors for the binding sites. The densities of the U3 and 1.4E modules of DLD-4 are colored transparent blue [36]. The densities of CRD2 and D1_401-560_ are overlaid onto the TcdB-DLD-4 complex and colored purple and pink, respectively. CRD2, cysteine-rich domain of frizzled-2; TcdB, Toxin B.
(PNG)

S1 Table Primer sequences for amplifying each of the genes. (PNG)

S2 Table Primers used in the mutagenesis for D1_410-560_. (PNG)

S3 Table Data collection and data processing of the cryo-EM density maps. (PNG)

S1 Movie 3D variability analysis of TcdB-CRD2 complex at pH 5. CRD2, cysteine-rich domain of frizzled-2; TcdB, Toxin B.
(MP4)

S2 Movie Morphing between the density maps obtained from focused classification around the D97 region of the TcdB-CRD2 complex (excluding the tip of the delivery domain and the tail of the CROPS domain) at pH 5. CRD2, cysteine-rich domain of frizzled-2; CROPS, combined repetitive oligopeptides; TcdB, Toxin B.
(MP4)

S3 Movie Morphing between the density maps obtained from focused classification around the D97 region of the TcdB-CRD2 complex (excluding the tip of the delivery domain and the tail of the CROPS domain) at pH 7.5. CRD2, cysteine-rich domain of frizzled-2; CROPS, combined repetitive oligopeptides; TcdB, Toxin B.
(MP4)

S1 Data Raw data for ELISA assays on interactions between TcdB mutants and D1_401-560_. The data correspond to Fig 2E. TcdB, Toxin B.
(XLSX)

S2 Data Raw data for ELISA assays on interactions between WT TcdB and D1_401-560_ mutants. The data correspond to Fig 2F. TcdB, Toxin B; WT, wild type.
(XLSX)

S3 Data Raw data for BLI measurements on interactions between TcdB and its receptors under pH 7.5 and pH 5. The data correspond to Fig 3A. BLI, bio-layer interferometry; TcdB, Toxin B.
(XLSX)

S4 Data Raw data for the resolution measurement of TcdB-D1_401-560_ complex. The data correspond to Panel D in S1 Fig. TcdB, Toxin B.
(XLSX)

S5 Data Raw data for the 1,000 D1_401-560_ models built from RosettaCM. The data correspond to Panel A in S3 Fig.
(XLSX)

S6 Data Data points for circular dichroism spectroscopy measurements of TcdB mutants. The data correspond to Panel A in S4 Fig. TcdB, Toxin B.
(XLSX)

S7 Data Data points for thermal shift measurements of D1_401-560_ mutants. The data correspond to Panel B in S4 Fig.
(XLSX)

S8 Data Raw data for ELISA assays on interactions between TcdB and its receptors under pH 7.5 and pH 5. The data correspond to S6 Fig. TcdB, Toxin B.
(XLSX)

S9 Data Raw data for the resolution measurement of the TcdB-CRD2 complex. The data correspond to Panel A in S7 Fig. CRD2, cysteine-rich domain of frizzled-2; TcdB, Toxin B.
(XLSX)

S10 Data Raw data for the resolution measurement of TcdB-CRD2 complex at pH 5. The data correspond to Panel B in S10 Fig. CRD2, cysteine-rich domain of frizzled-2; TcdB, Toxin B.
(XLSX)

## Abbreviations

APD: autoprocessing domain
BLI: bio-layer interferometry
CDI: *C*. *difficile* infection
CRD: cysteine-rich domain
CRD2: cysteine-rich domain of frizzled-2
CROPS: combined repetitive oligopeptides
CSPG4: chondroitin sulfate proteoglycan 4
CTF: contrast transfer function
D1: Domain 1
D2: Domain 2
D3: Domain 3
FAK: focal adhesion kinase
FSC: Fourier shell correlation
FZD2: frizzled-2
GFP: green fluorescent protein
GTD: glucosyltransferase domain
LGR: laminin G-type region
PAM: palmitoleic acid
TcdB: Toxin B
