# Supplementary figures and images for: Structural dynamics of receptor recognition and pH-induced dissociation of full-length Clostridioides difficile Toxin B
Source: PLoS Biol. 2022 Mar 24;20(3):e3001589. doi: 10.1371/journal.pbio.3001589 (PMC8982864; doi:10.1371/journal.pbio.3001589)

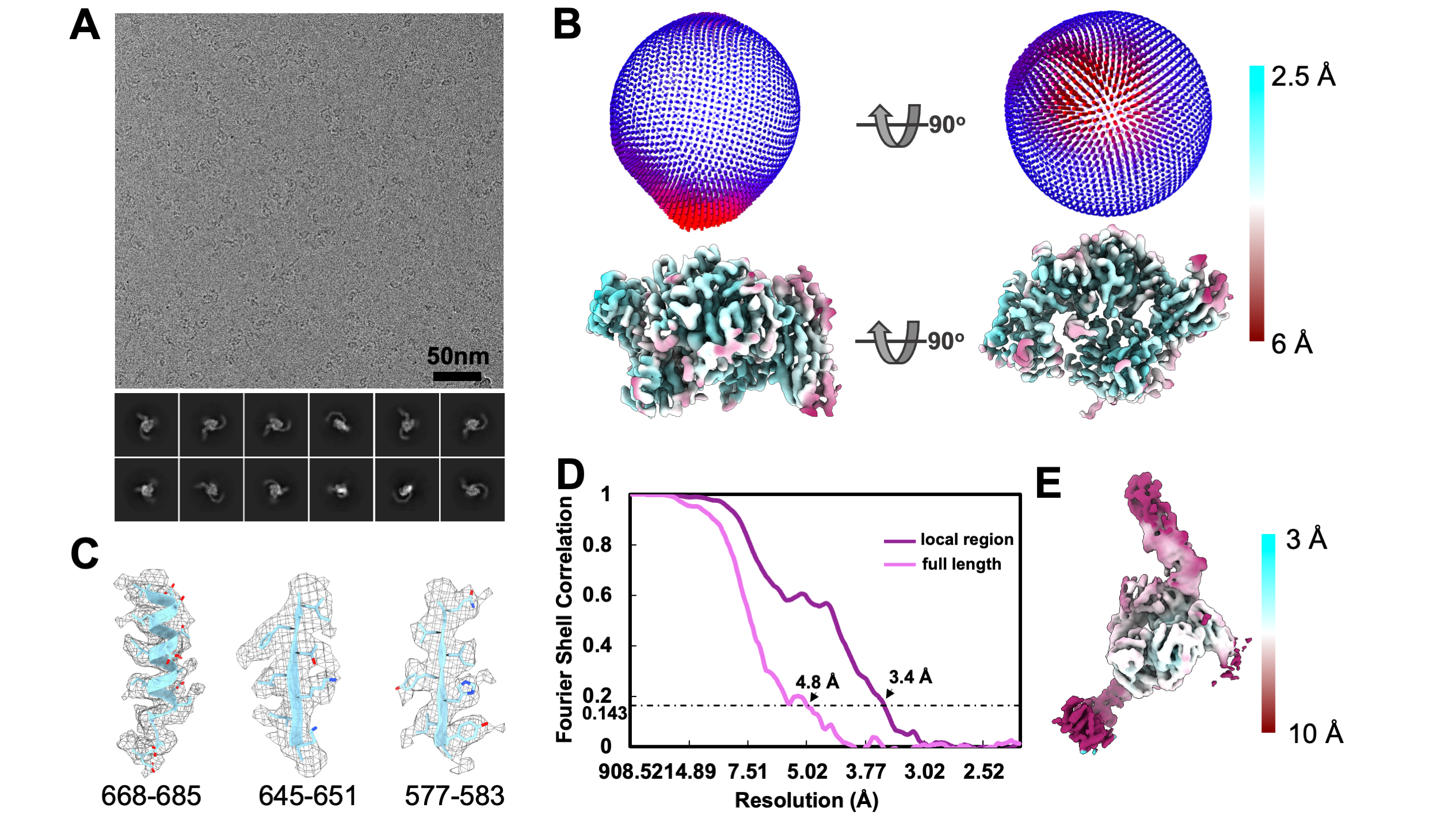

Supplement: S1 Fig — (A) Representative micrograph and 2D classifications of the TcdB-D1401-560 complex. (B) The orientation distribution (top) and local resolution (bottom) for the reconstruction of the central region of the TcdB-D1401-560 complex. (C) The representative densities show bulky side chains. (D) FSCs of the cryo-EM reconstructions (S4 Data). (E) The local resolution for the reconstruction of the complete TcdB-D1401-560 complex. FSC, Fourier shell correlation; TcdB, Toxin B. (PNG) [file pbio.3001589.s001.png]

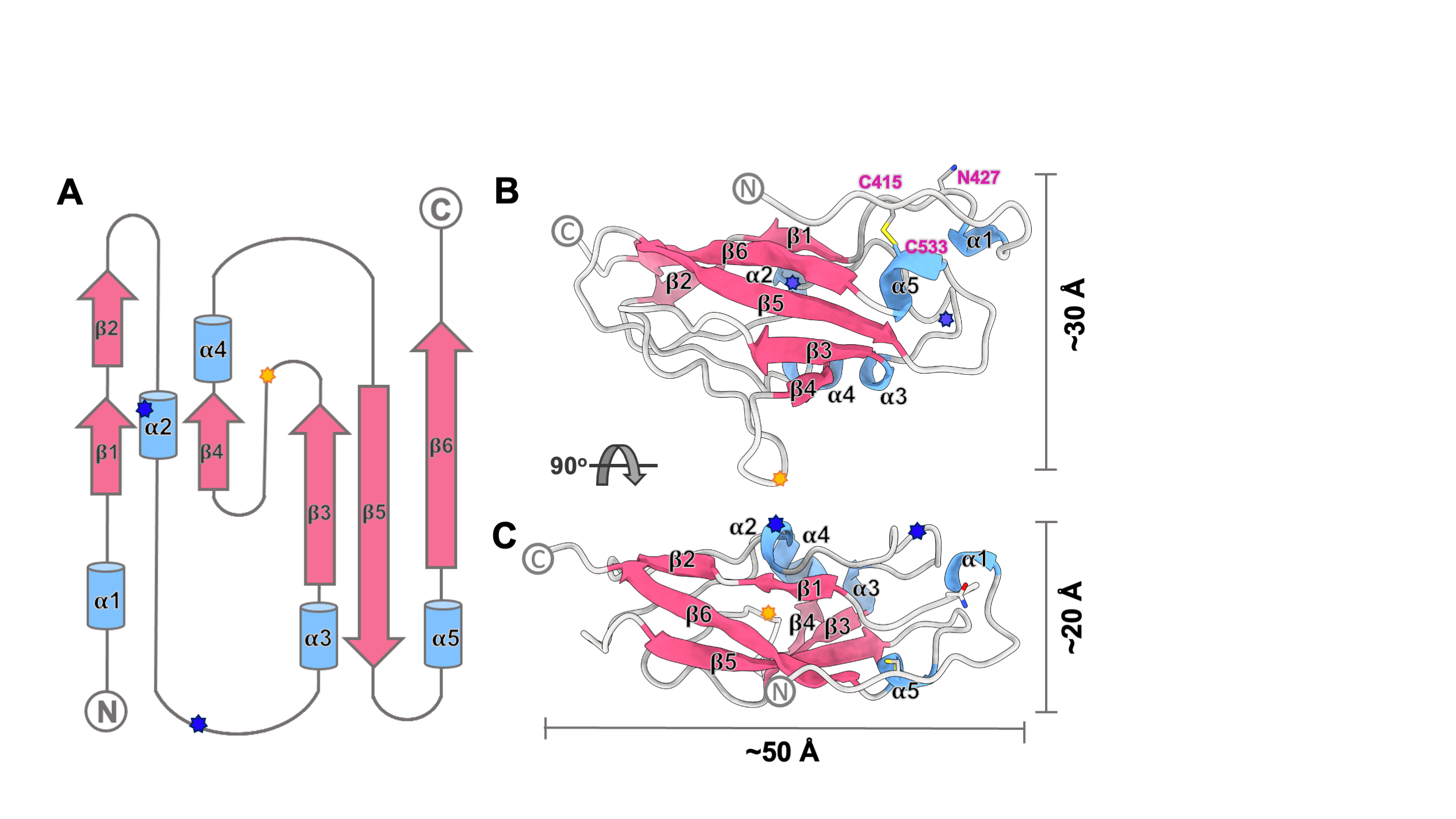

Supplement: S2 Fig — (A) The topology of D1401-560 with α-helices and β-strands shown as blue cylinders and red arrows, respectively. The N- and C-termini are labeled by circles. The blue stars and the yellow star highlight the binding regions for APD and the delivery domain of TcdB, respectively. (B) The atomic model of D1401-560. A disulfide bond is present and residue N427 for glycosylation is labeled. (C) The model of D1401-560 rotated 90° from the view in Panel B. The dimensions of D1401-560 are labeled. APD, autoprocessing domain; TcdB, Toxin B. (PNG) [file pbio.3001589.s002.png]

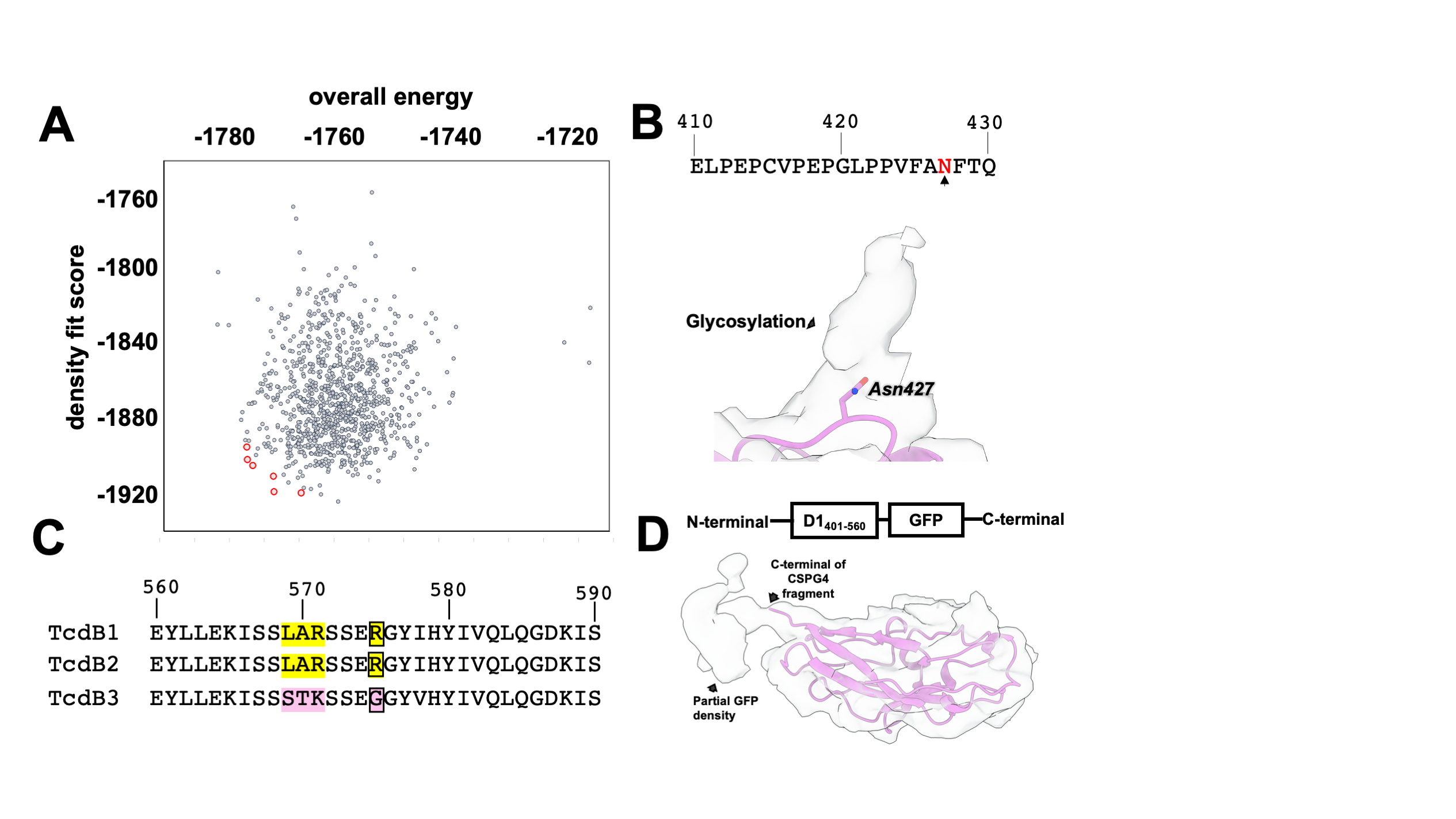

Supplement: S3 Fig — (A) The diagram shows the overall energy and the EM density fit score for 1,000 models of D1401-560 generated by RosettaCM [48]. The 6 chosen models were labeled in red circles (S5 Data). (B) The glycosylation site was predicted through the webserver NetNGlyc-1.0 [27] and highlighted in red (top). The density of the potential glycan on Asn427 of D1401-560 is shown (bottom). (C) The primary sequence alignment among TcdB1, TcdB2, and TcdB3 from residue 560 to residue 590. The sequence differences were highlighted. The residue 575 of the 3 TcdB subtypes were boxed. (D) Extra density for the GFP tag indicates the position of the D1401-560 C-terminus. CSPG4, chondroitin sulfate proteoglycan 4; GFP, green fluorescent protein; TcdB, Toxin B. (PNG) [file pbio.3001589.s003.png]

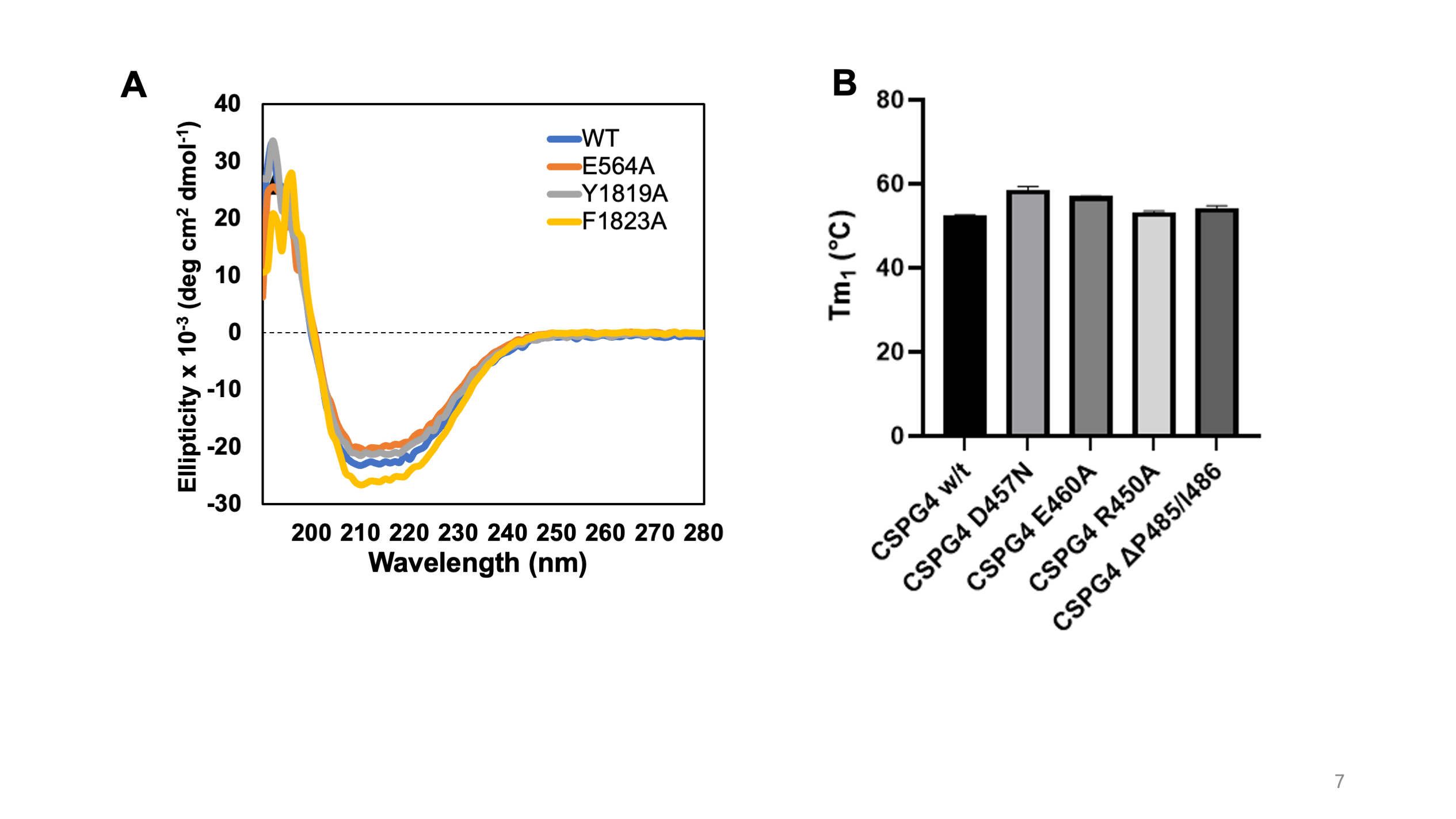

Supplement: S4 Fig — (A) Representative circular dichroism spectroscopy on TcdB mutants. Measurements were repeated twice and produced similar results (S6 Data). (B) The thermal stability of mutant D1401-560 of CSPG4 proteins were measured using label-free, native differential scanning fluorimetry (nanoDSF; Prometheus NT.48, NanoTemper). The data are presented as means ± SD (n = 3). D1401-560 variants showed Tm1 values comparable to the wild-type D1401-560 protein, which implies the correct protein folding (S7 Data). CSPG4, chondroitin sulfate proteoglycan 4; TcdB, Toxin B; WT, wild type. (PNG) [file pbio.3001589.s004.png]

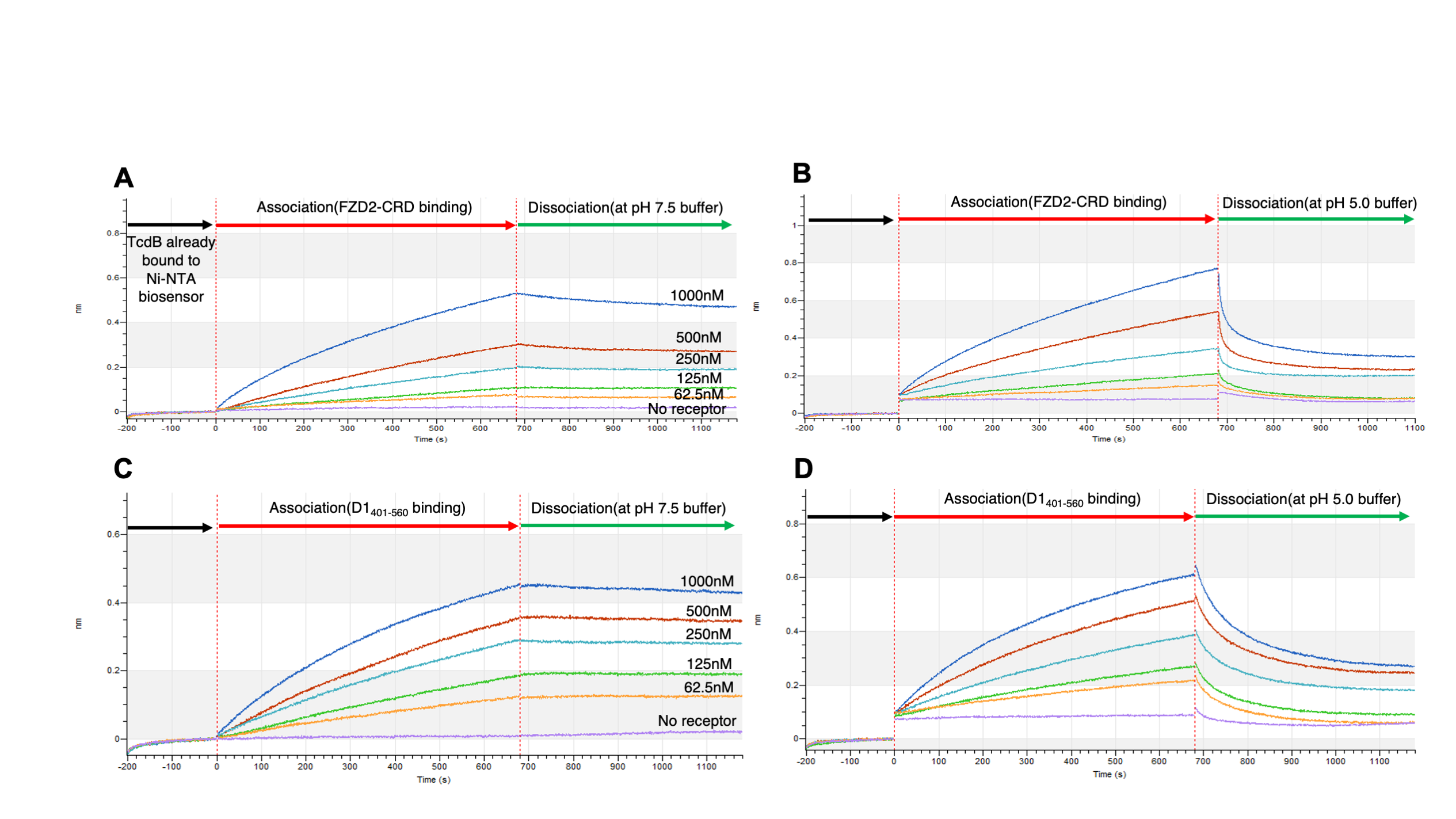

Supplement: S5 Fig — Representative binding curves of 4 different conditions after TcdB, as a ligand, are immobilized on Ni-NTA biosensors. The concentrations of the receptor proteins (FZD2-CRD, D1401-560) are labeled in each panel. The reference wells, which assay buffer was added instead of receptor proteins in association step, are depicted in purple. (A) pH 7.5 dissociation after CRD2 binding. (B) pH 5.0 dissociation after CRD2 binding. (C) pH 7.5 dissociation after D1401-560 binding. (D) pH 5.0 dissociation after D1401-560 binding. BLI, bio-layer interferometry; CRD2, cysteine-rich domain of frizzled-2; FZD2, frizzled-2; TcdB, Toxin B. (PNG) [file pbio.3001589.s005.png]

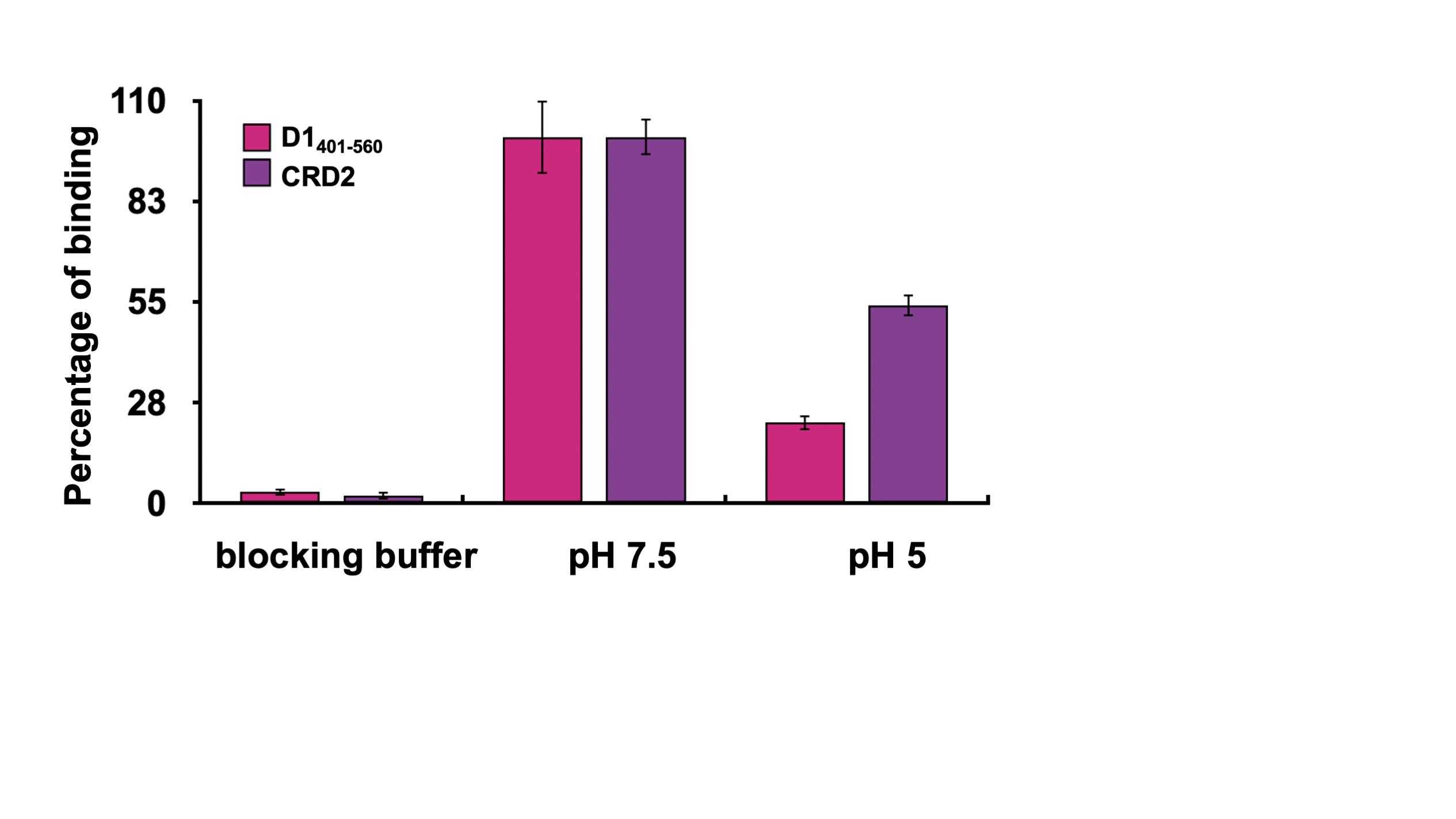

Supplement: S6 Fig — The signals of bound D1401-560/CRD2 at pH 7.5 were scaled to the same level and defined as 100% binding (S8 Data). Error bars represent standard deviations (n = 2). CRD2, cysteine-rich domain of frizzled-2; TcdB, Toxin B. (PNG) [file pbio.3001589.s006.png]

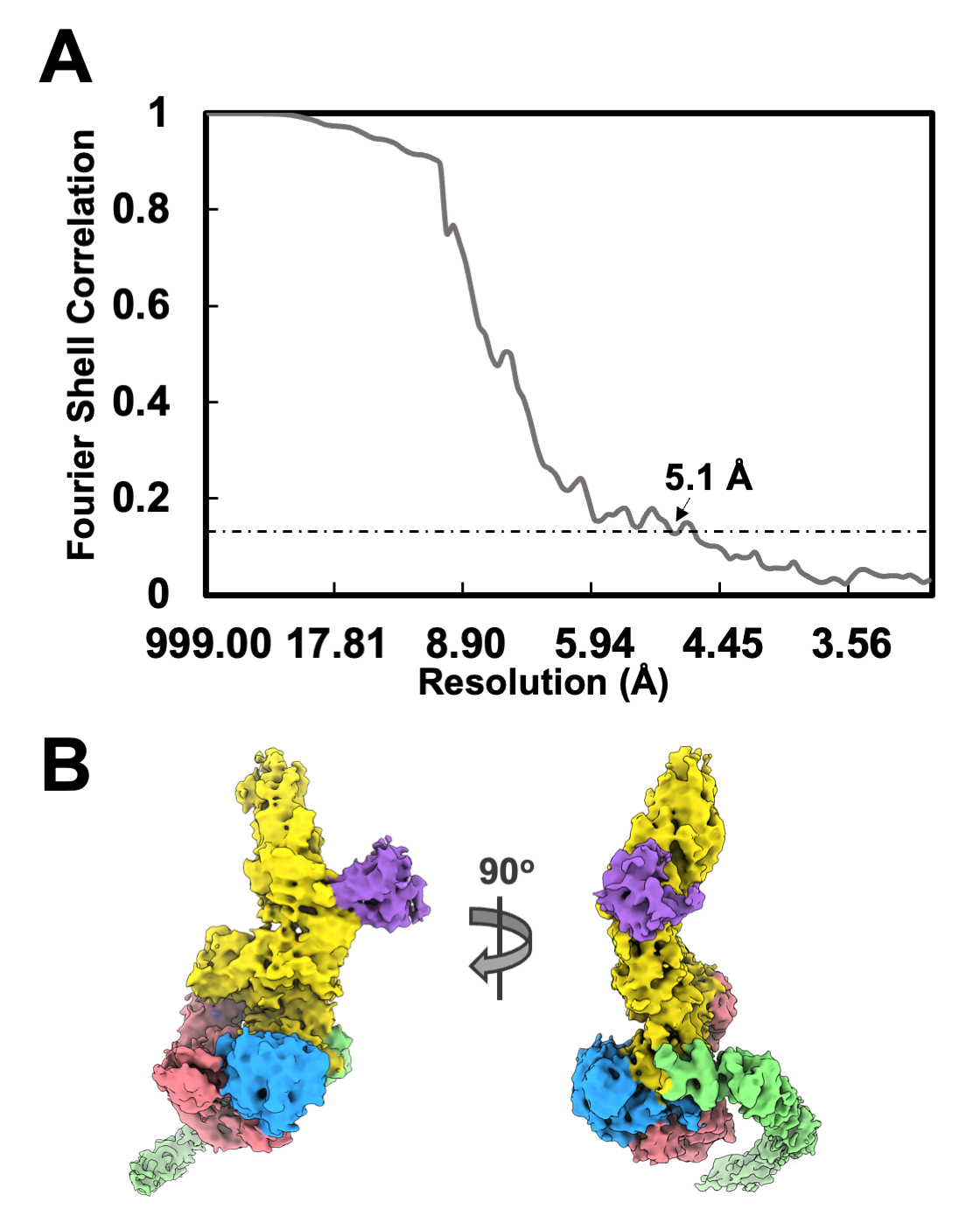

Supplement: S7 Fig — (A) The FSC of the cryo-EM reconstruction (S9 Data). (B) Two views of the TcdB-CRD2 density map at pH 7.5. Each TcdB domain was colored based on the scheme in Fig 1. The CRD of FZD2 (or CRD2) is colored purple. CRD, cysteine-rich domain; CRD2, cysteine-rich domain of frizzled-2; FSC, Fourier shell correlation; FZD2, frizzled-2; TcdB, Toxin B. (PNG) [file pbio.3001589.s007.png]

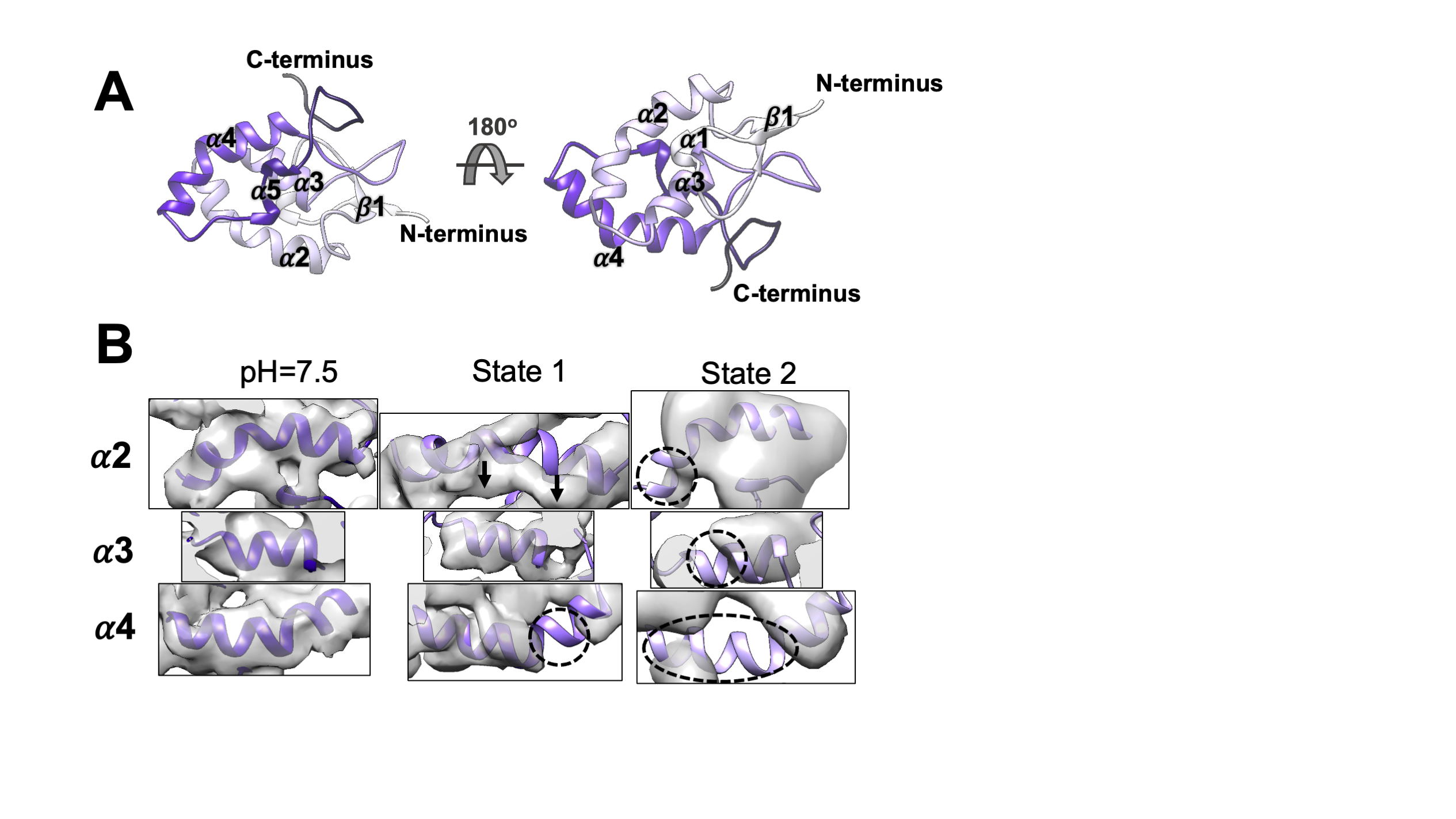

Supplement: S8 Fig — (A). The model of the CRD2 with secondary structures labeled. (B). The model of CRD2 (purple ribbons) from the structure of the TcdB-CRD2 complex, at pH 7.5, was docked into the density maps (transparent gray) in state 1 and state 2. The fitting of ɑ2, ɑ3, and ɑ4 in the densities for the pH 7.5 state, pH 5 State 1, and pH 5 State 2 are shown. The proposed unfolded regions were marked by black dashed circles. All the CRD2 densities were kept at the same surface area level for comparison (approximately 7,400 Å2). CRD2, cysteine-rich domain of frizzled-2; TcdB, Toxin B. (PNG) [file pbio.3001589.s008.png]

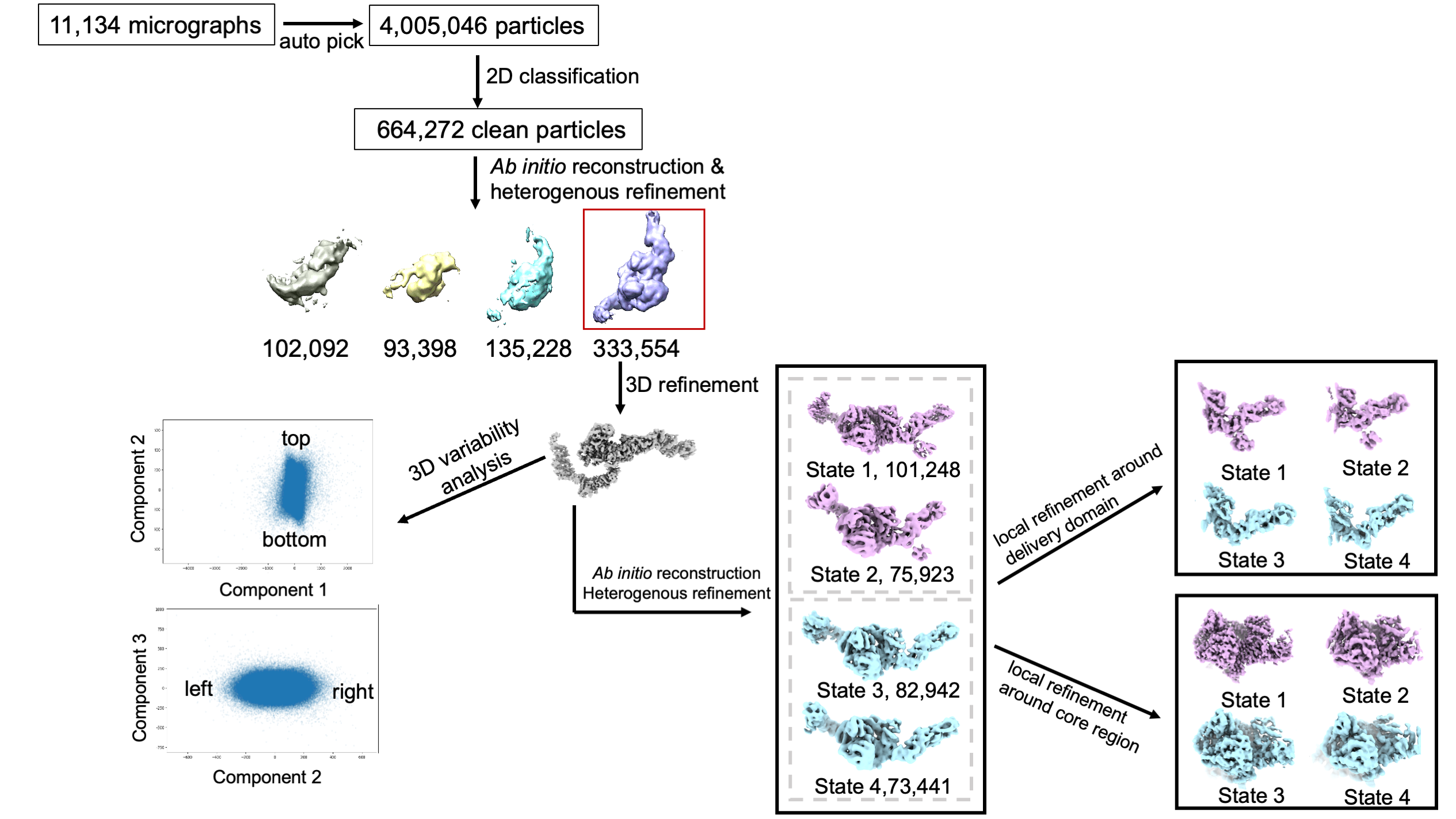

Supplement: S9 Fig — See Methods for details. In brief, 4,005,046 particles were autopicked from 11,134 motion corrected micrographs. The picked particles were further cleaned using 2D classification, ab initio reconstruction, and heterogeneous refinement. Only one class of heterogeneous refinement generated a good density map. A total of 333,554 particles from this class were used for consensus 3D refinement followed by 3D variability analysis. The variability analysis showed that the TcdB-CRD2 complex at pH 5 exhibited continuous conformational changes. Based on the principal components 1–3, we were able to distinguish the particles into roughly 4 conformations (top and bottom based on Components 1 and 2, left and right based on Components 2 and 3). Therefore, in the second round of heterogeneous refinement, we requested for 4 states of the TcdB-CRD2 complex at pH 5. These 4 classes were used as initial models for further refinement of each class. Local refinement was then applied by using a mask around the delivery domain or core region to obtain higher-resolution densities for model building. CRD2, cysteine-rich domain of frizzled-2; TcdB, Toxin B. (PNG) [file pbio.3001589.s009.png]

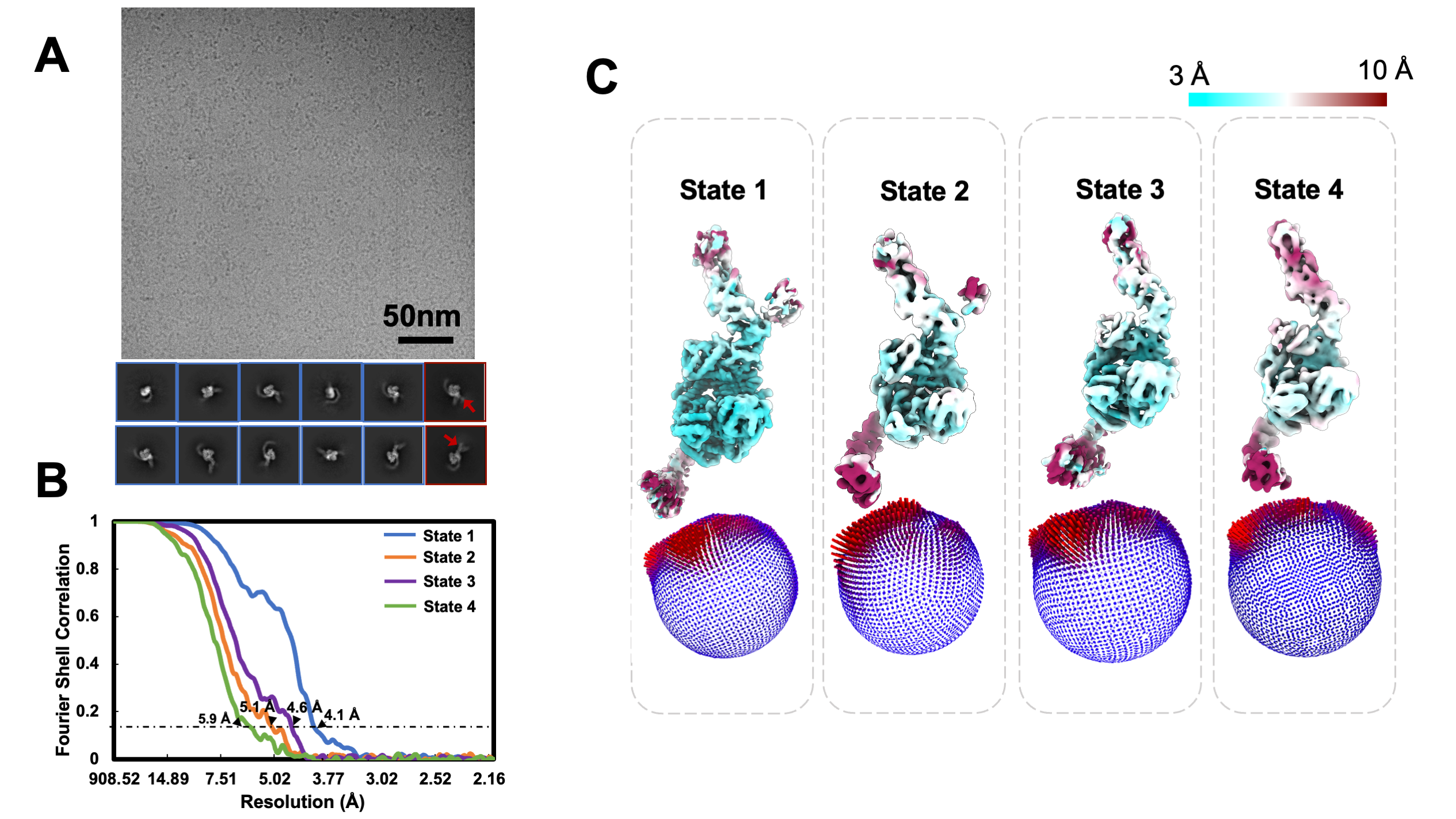

Supplement: S10 Fig — (A) A representative micrograph and 2D classifications of the TcdB-CRD2 complex at pH 5. The projections with CRD2 density were highlighted with red boxes. The binding position was illustrated by red arrows. (B) FSCs of the 4 cryo-EM reconstructions of the TcdB-CRD2 complex at pH 5 (S10 Data). (C) Local resolutions (top) and orientation distributions (bottom) of 4 states of the TcdB-CRD2 at pH 5. All the maps were displayed at the same contour level (0.033) with cyan for high resolutions and maroon for low resolutions. CRD2, cysteine-rich domain of frizzled-2; FSC, Fourier shell correlation; TcdB, Toxin B. (PNG) [file pbio.3001589.s010.png]

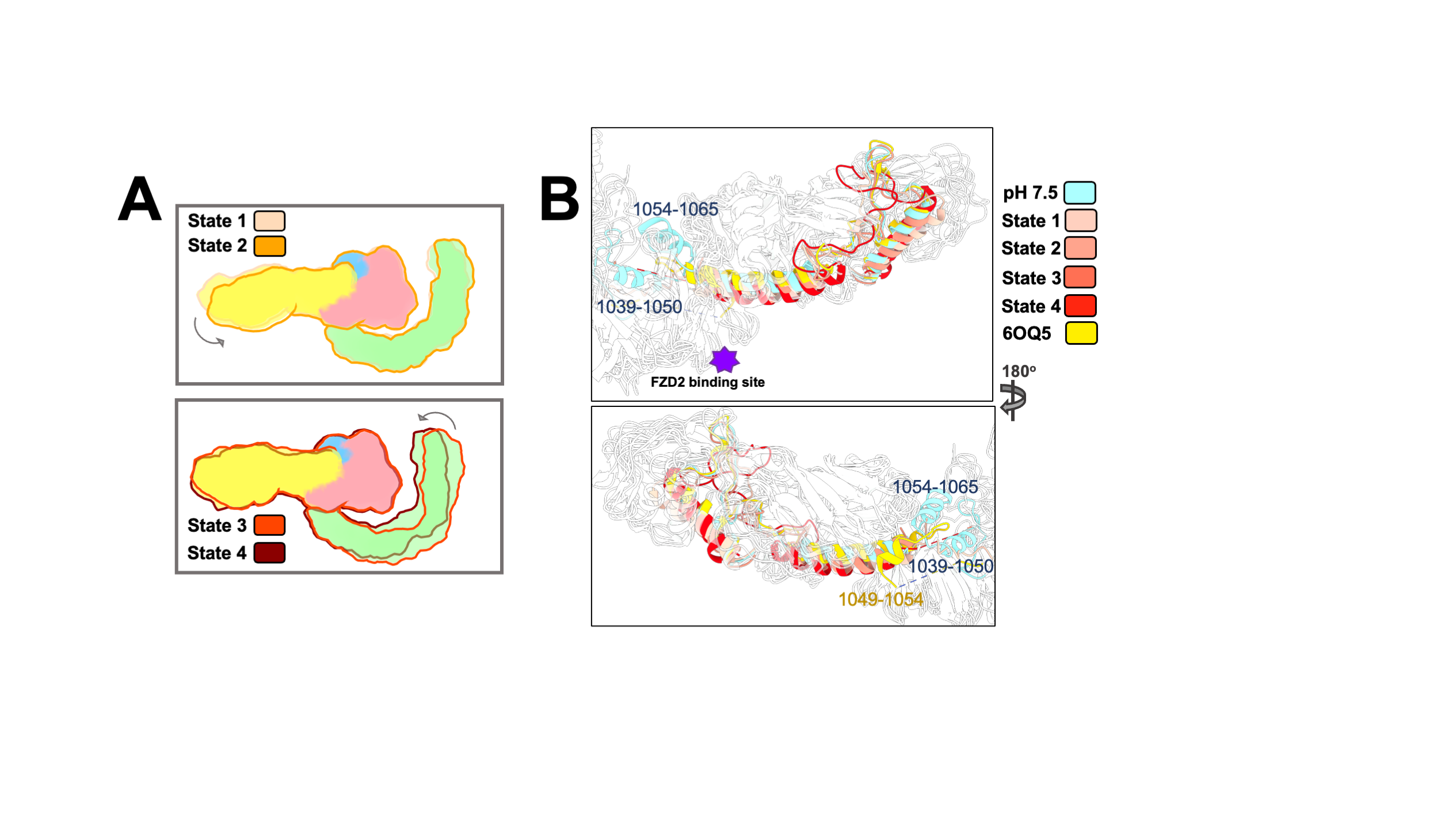

Supplement: S11 Fig — (A) The scheme of domain movements between states at pH 5. TcdB domains are colored based on the scheme in Fig 1. (B) The rearrangement of the pore-forming helices from pH 7.5 (blue ribbon model) to pH 5 (ribbon models with different shades of red), as well as the crystal structure of TcdB at pH 5 (yellow: PDB: 6OQ5) [9]. At pH 5, helices consist of residues 1,039–1,050, and residues 1,054–1,065 become flexible and unresolved. The structures are aligned based on the delivery domain. As in our cryo-EM structures at pH 5, the X-ray structure also has helices 1,039–1,050 and 1,054–1,065 disordered. Interestingly, residues 1,049–1,054, which was originally a loop connecting 2 helices at pH 7, has changed into a short helical structure and flipped outside the core of the delivery domain, potentially for the membrane insertion. FZD2, frizzled-2; TcdB, Toxin B. (PNG) [file pbio.3001589.s011.png]

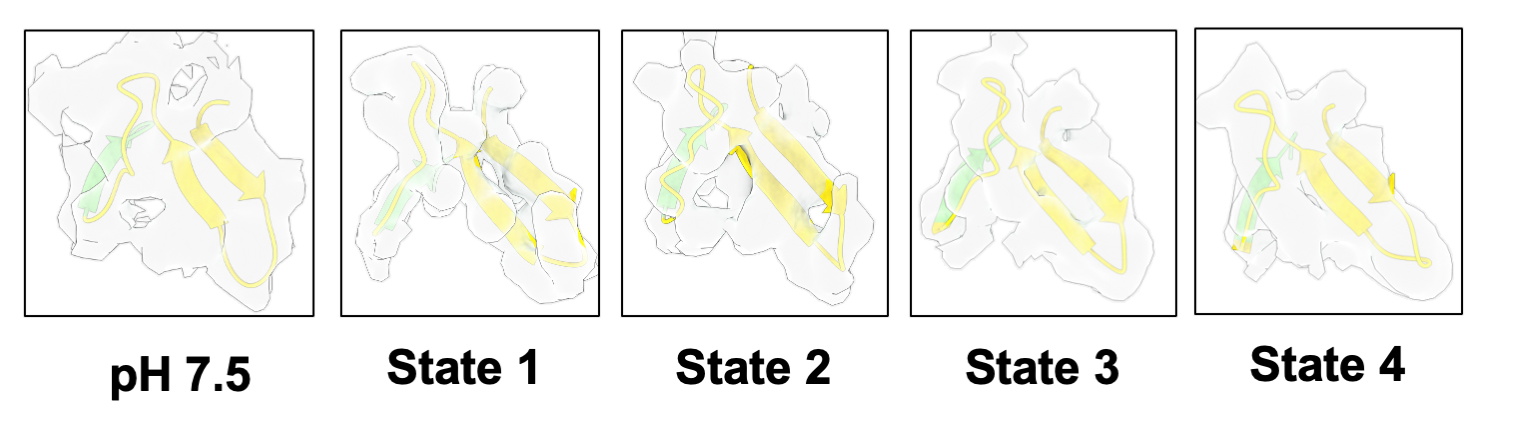

Supplement: S12 Fig — (PNG) [file pbio.3001589.s012.png]

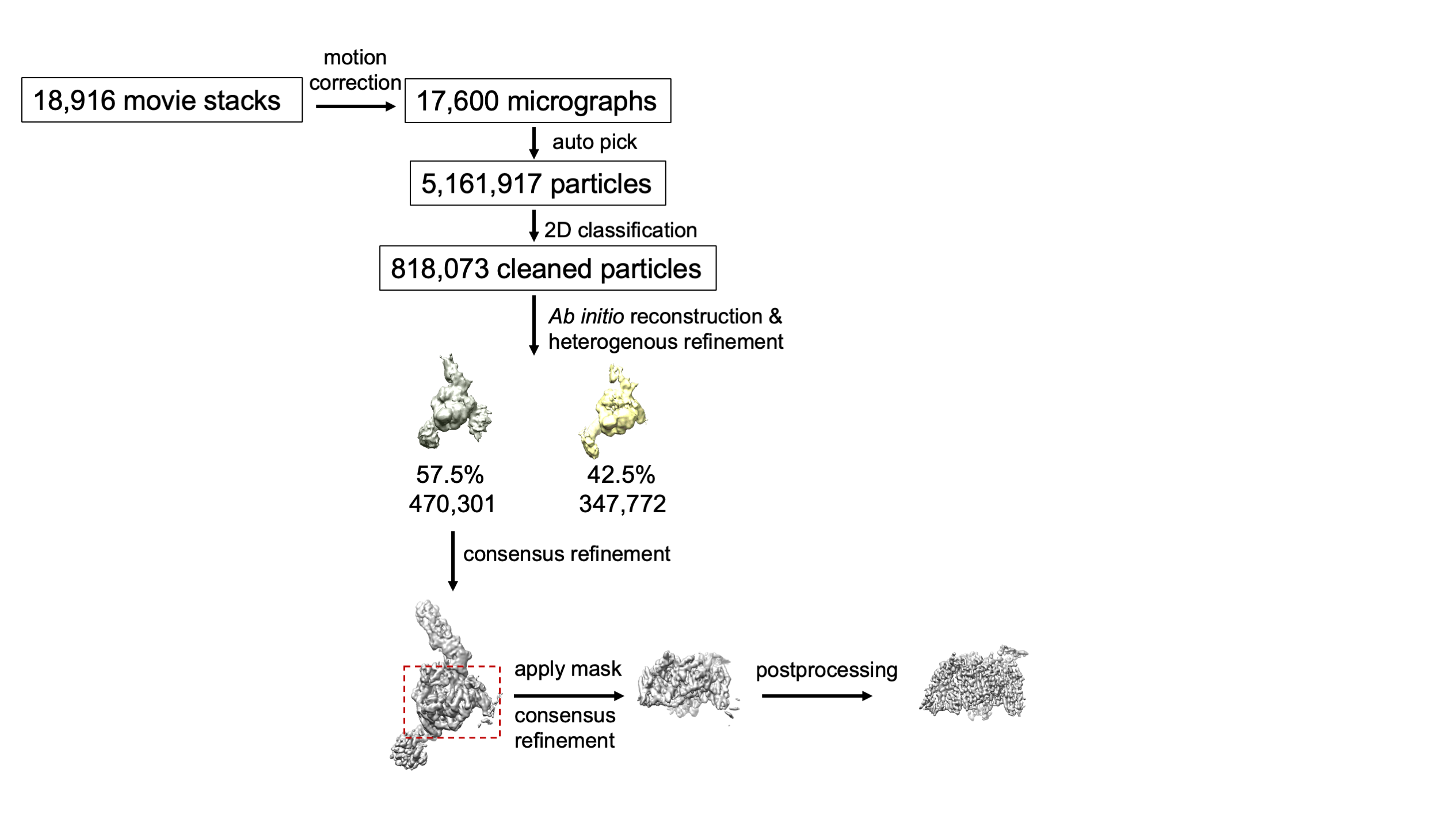

Supplement: S13 Fig — The details are provided in Methods. (PNG) [file pbio.3001589.s013.png]

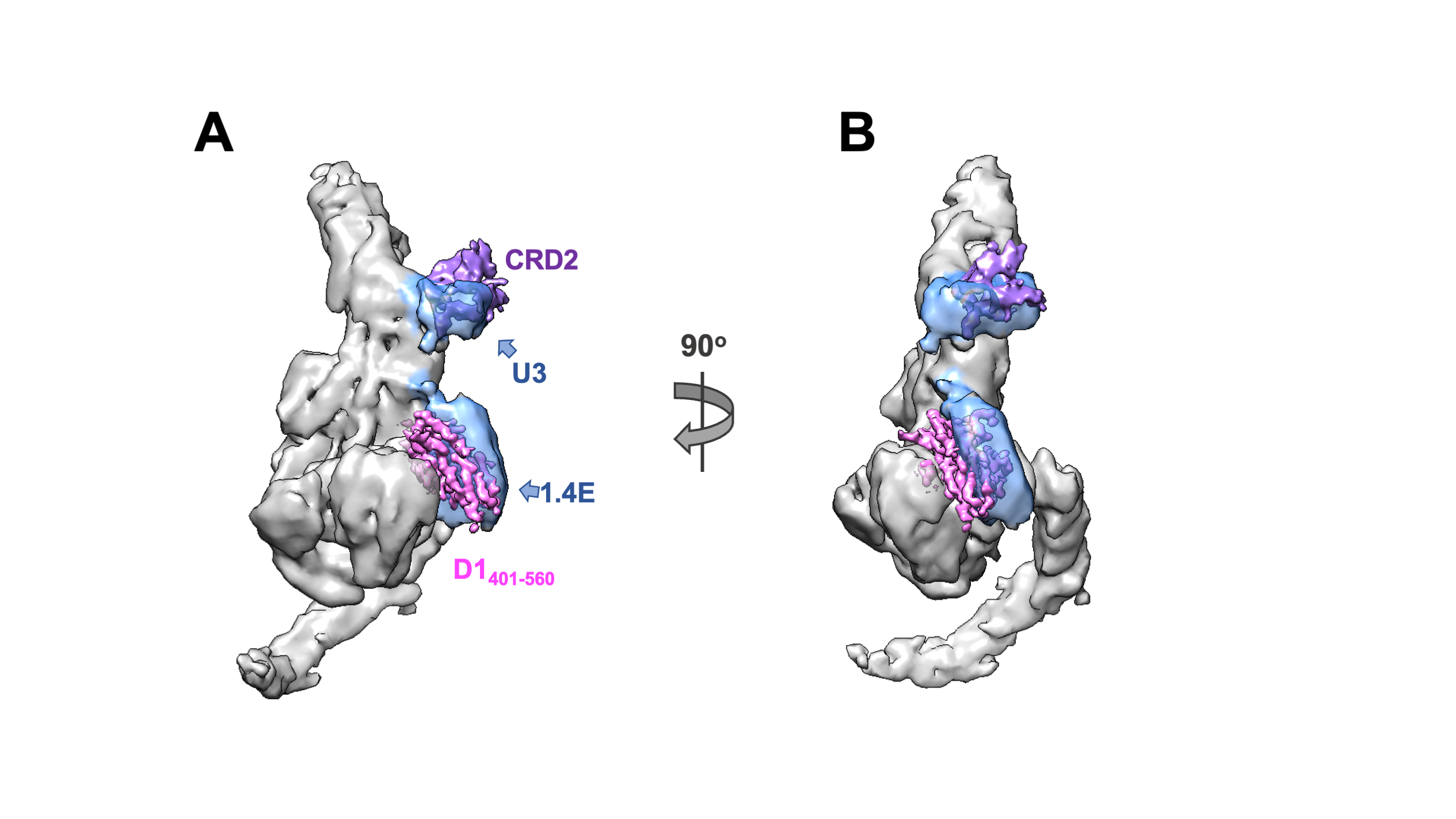

Supplement: S14 Fig — The densities of the U3 and 1.4E modules of DLD-4 are colored transparent blue [36]. The densities of CRD2 and D1401-560 are overlaid onto the TcdB-DLD-4 complex and colored purple and pink, respectively. CRD2, cysteine-rich domain of frizzled-2; TcdB, Toxin B. (PNG) [file pbio.3001589.s014.png]

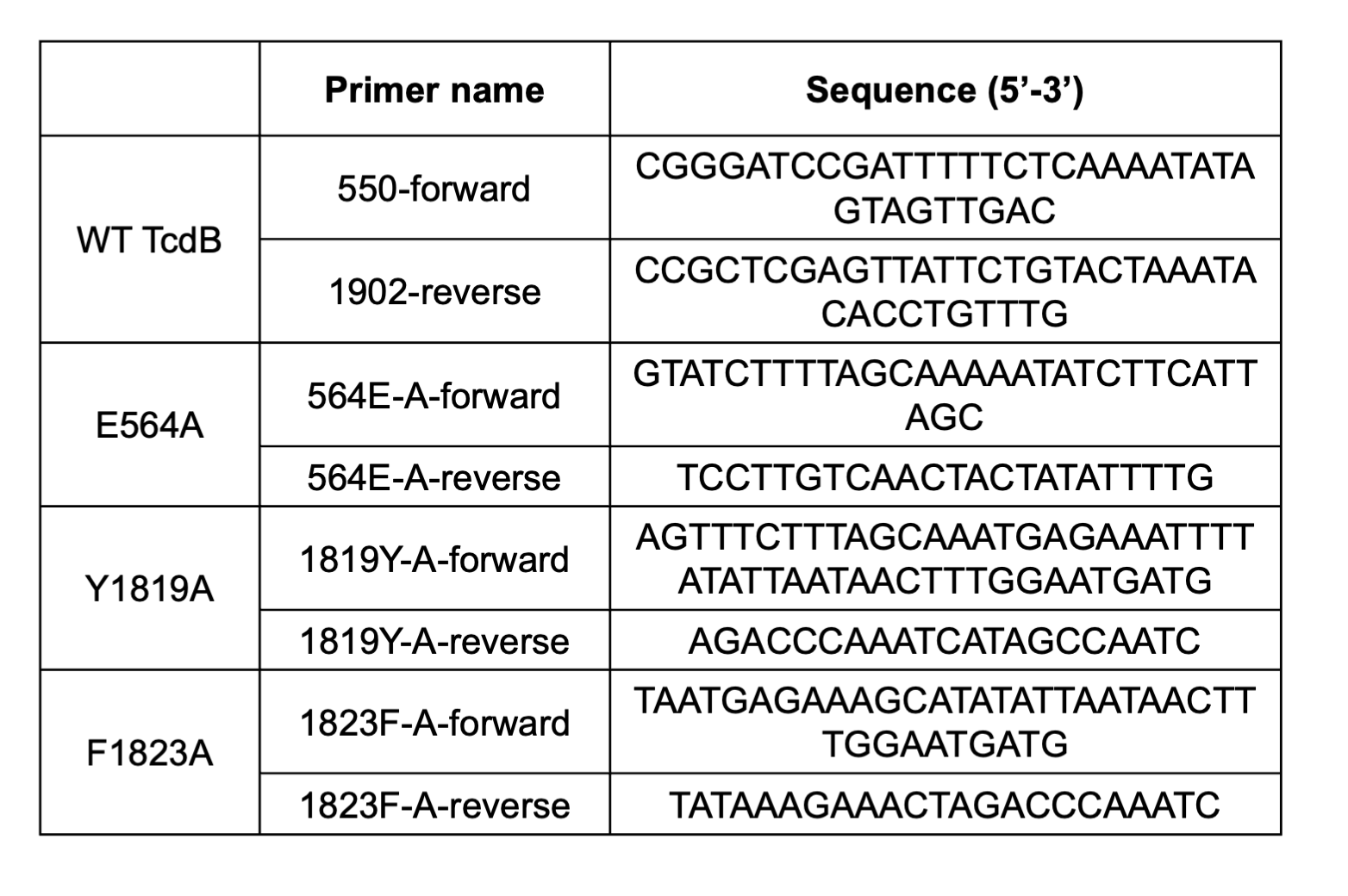

Supplement: S1 Table — (PNG) [file pbio.3001589.s015.png]

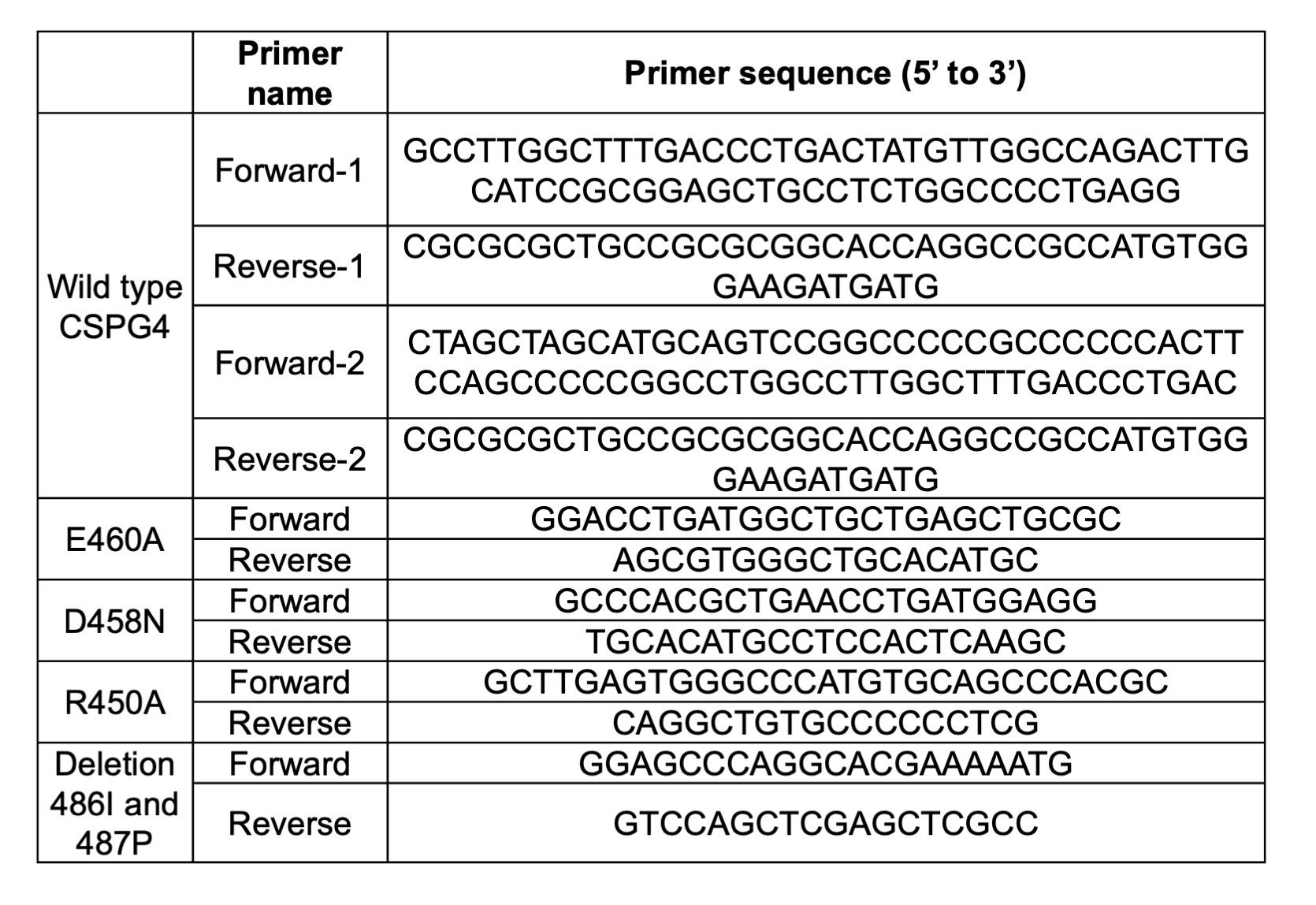

Supplement: S2 Table — (PNG) [file pbio.3001589.s016.png]

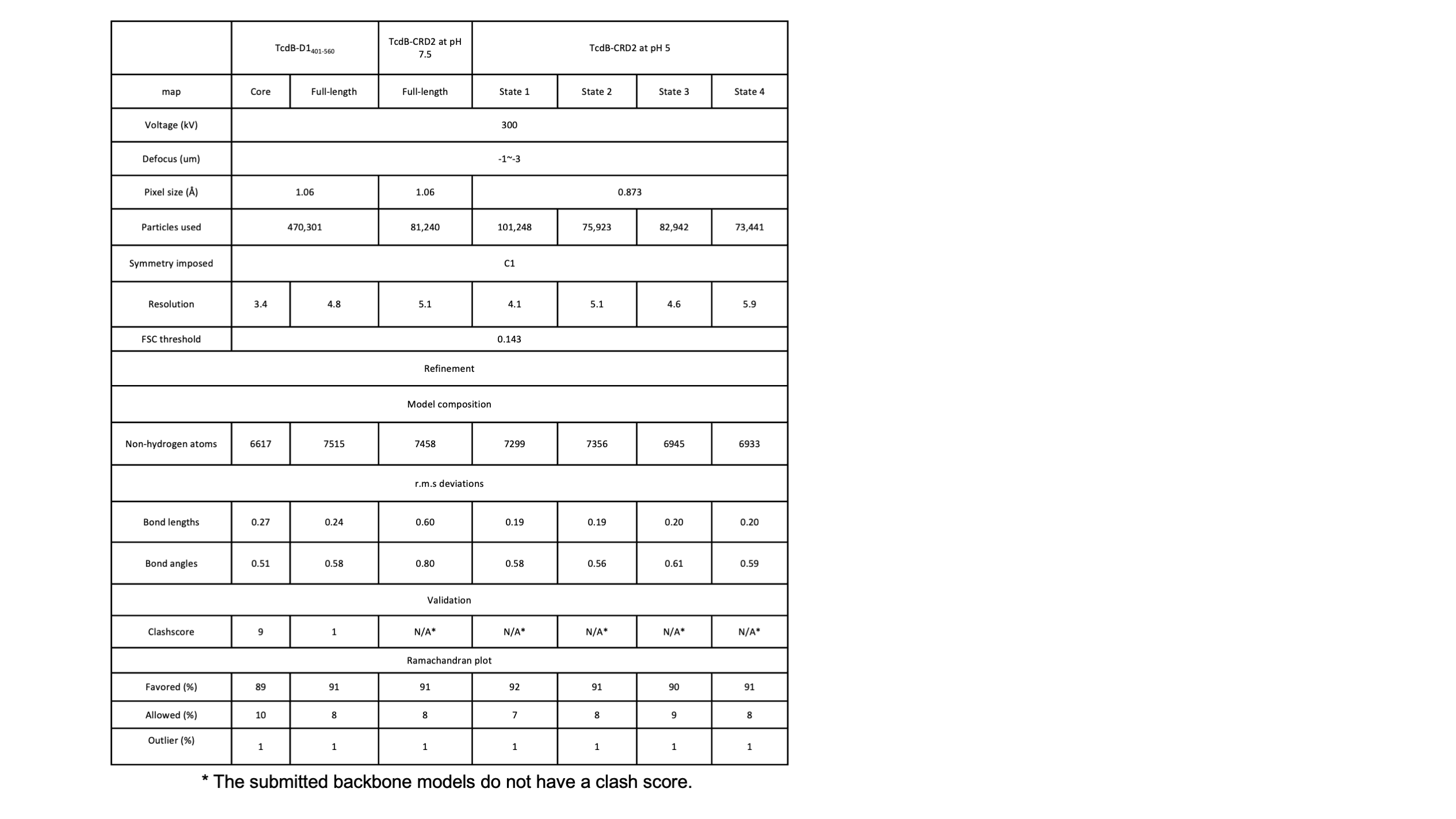

Supplement: S3 Table — (PNG) [file pbio.3001589.s017.png]
